# Supplementary material for: Heterogeneity in oligodendrocyte precursor cell proliferation is dynamic and driven by passive bioelectrical properties
Source: Cell Rep. 2024 Oct 17;43(11):114873. doi: 10.1016/j.celrep.2024.114873 (PMC11602547; doi:10.1016/j.celrep.2024.114873)
Supplement: Document S1. Figures S1–S7 [file mmc1.pdf]

Cell Reports, Volume *43*

Supplemental information

Heterogeneity in oligodendrocyte precursor cell proliferation is dynamic and driven by passive bioelectrical properties

Helena Pivoňková, Sergey Sitnikov, Yasmine Kamen, An Vanhaesebrouck, Moritz Matthey, Sonia Olivia Spitzer, Yan Ting Ng (吳胤霆), Chenyue Tao (陶辰玥), Omar de Faria Jr., Balazs Viktor Varga, and Ragnhildur Thóra Káradóttir

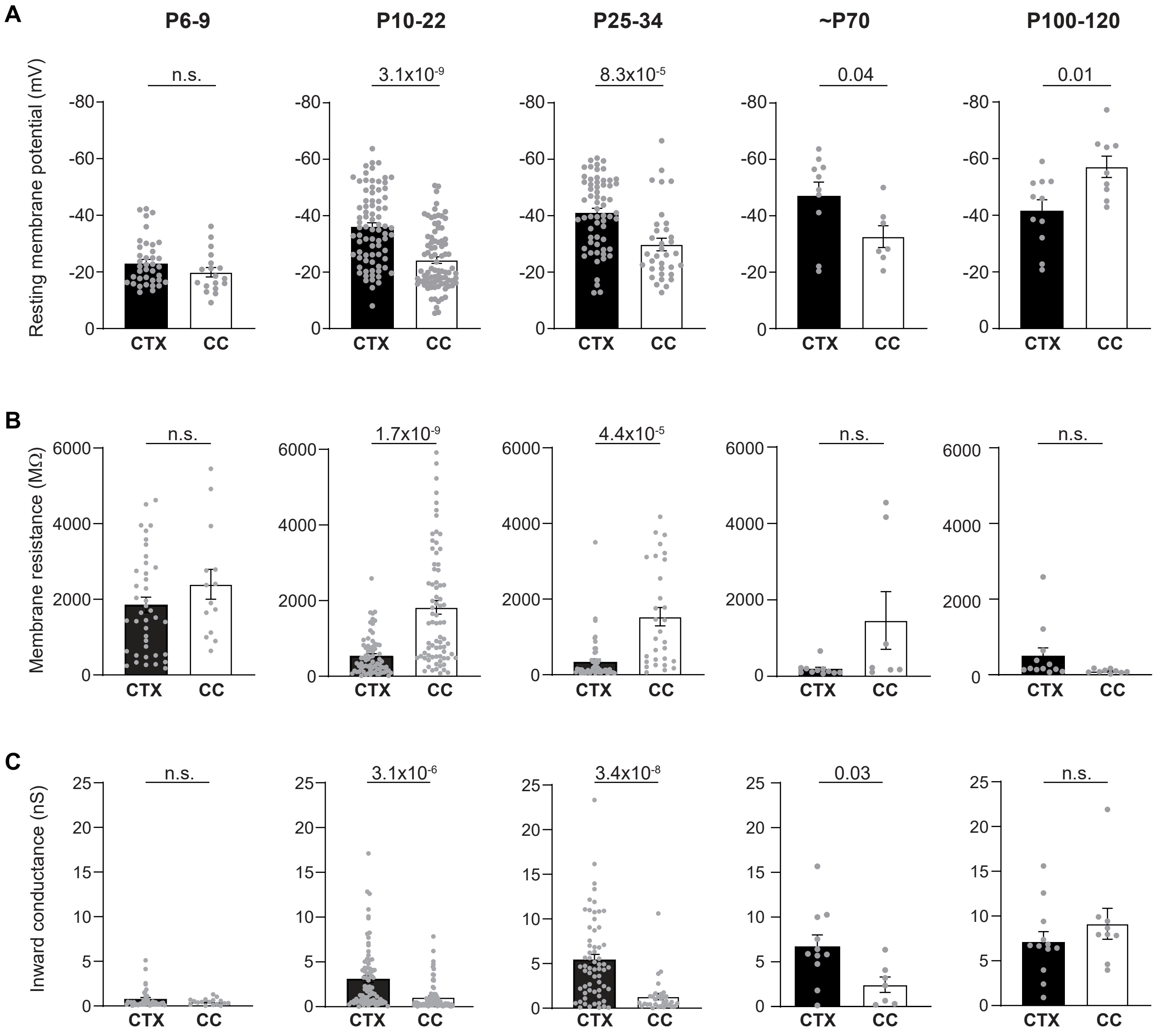

Figure S1. Membrane properties comparison between cortical and callosal OPCs at different ages, related to Figure 1.

(**A**) Resting membrane potential, (**B**) membrane resistance and (**C**) inward conductance are compared between cortical and callosal OPCs at different ages.

Individual grey dots indicate individual recorded cells. p values were calculated using unpaired two-tailed t-tests or t-tests with Welch's correction. Values are given as mean ± s.e.m..

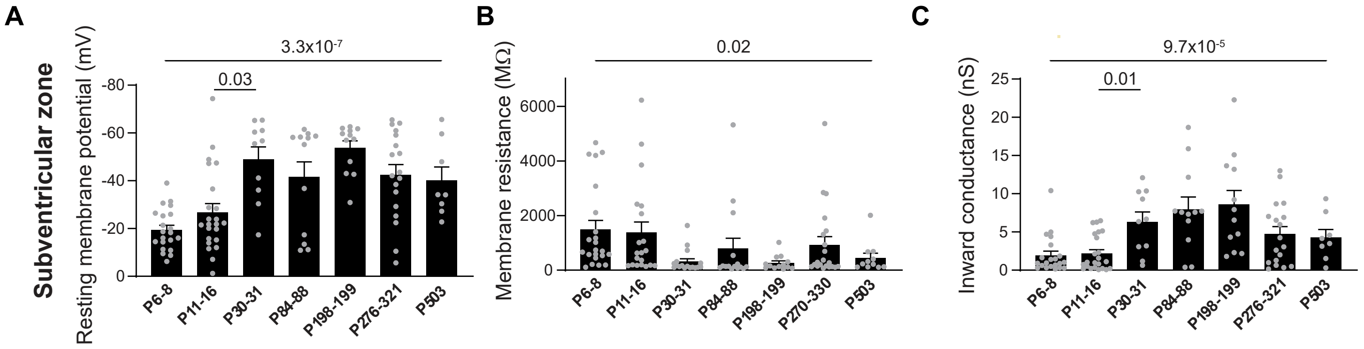

Figure S2. Membrane properties of OPCs in the subventricular zone, related to Figure 1.

(**A**) Resting membrane potential, (**B**) membrane resistance and (**C**) inward conductance were recorded in EYFP$^+$ cells in NG2-EYFP mice in the subventricular zone at different ages.

Grey dots represent individual recorded cells. p values were calculated using one-way ANOVA (top) with Holm-Bonferroni post-hoc tests (bottom). Values are given as mean ± s.e.m..

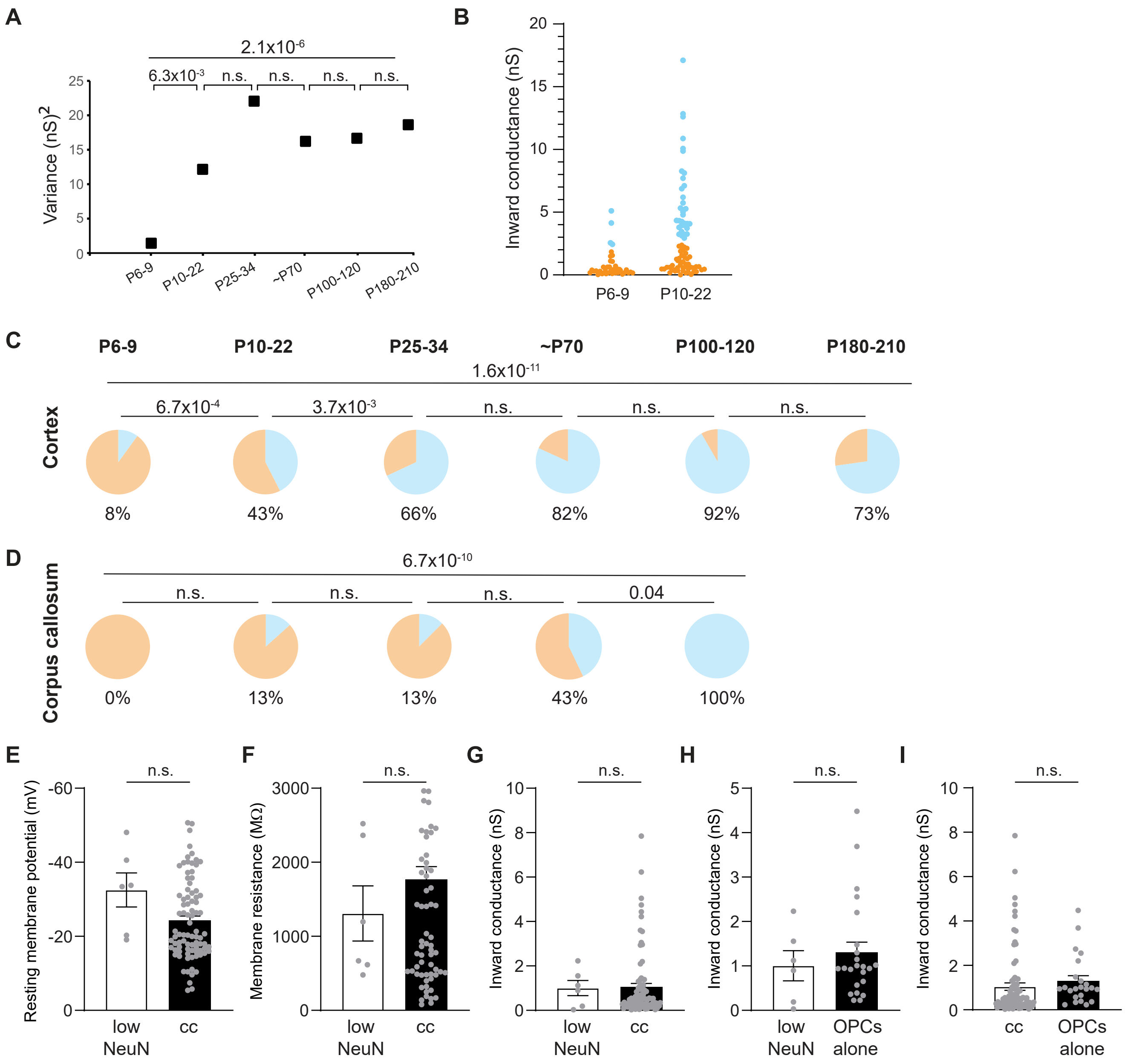

Figure S3. OPC subgroups based on inward conductance variability, related to Figure 2.

(**A**) Variance in cortical OPC inward conductance at different ages. p values of the differences in the variability between age groups were calculated using Levene's variability multi-comparison test.

(**B**) Inward conductance in OPCs in young animals clearly show separation into two groups, OPCs with low conductance (orange) and OPCs with high conductance (blue), the number of which increases with age.

(**C-D**) Percentages of OPCs with low (orange) and high (blue) inward conductance in different age groups in the cortex (**C**) and corpus callosum (**D**). OPCs were assigned into high conductance group when the inward conductance was higher than 2.35 nS as calculated from the 90th percentile of the two Gaussian curves (see the results and Figure 2). The percentages below the pie charts indicate the proportion of OPCs with high inward conductance. p values were calculated with χ^2- test (top) and χ^2- test with Yates correction (bottom).

(**E-I**) OPCs in areas of low neuronal density have similar membrane properties. The resting membrane potential (**E**), membrane resistance (**F**), and inward conductance (**G**) in cortical OPCs in areas of low neuronal density are similar to age-matched passive membrane properties of OPCs in the corpus callosum. OPCs in low neuronal density cortical areas and in the corpus callosum have similar inward conductance to OPCs cultured without neurons (**H,I**).

Dots represent individual recorded cells. p values are from two-tailed unpaired t-test or t-test with Welch's correction. Data are presented as mean ± s.e.m..

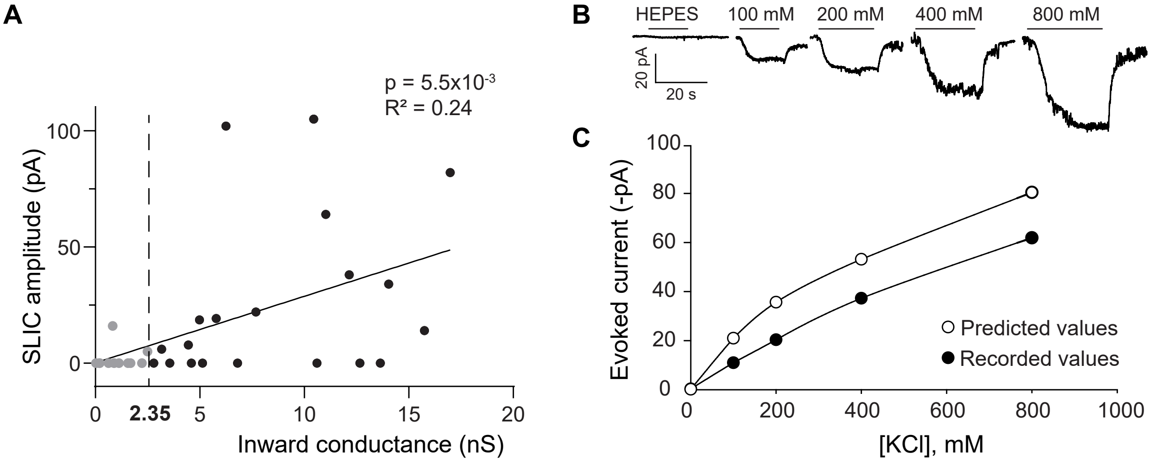

Figure S4. OPCs respond to increases in $[K^+]_e$ by increasing the inward current, related to Figure 4.

(**A**) The amplitude of SLICs correlate with the inward conductance in OPCs. Dots represent individual recorded cells. p value is from linear regression analysis.

(**B**) Increasing the concentration of K^+ in aCSF leads to proportional increase in the inward current in OPCs.

(**C**) The recorded inward current in OPCs evoked by increasing concentration of K^+ in aCSF follows the current predicted from the Nernst equation (see Methods).

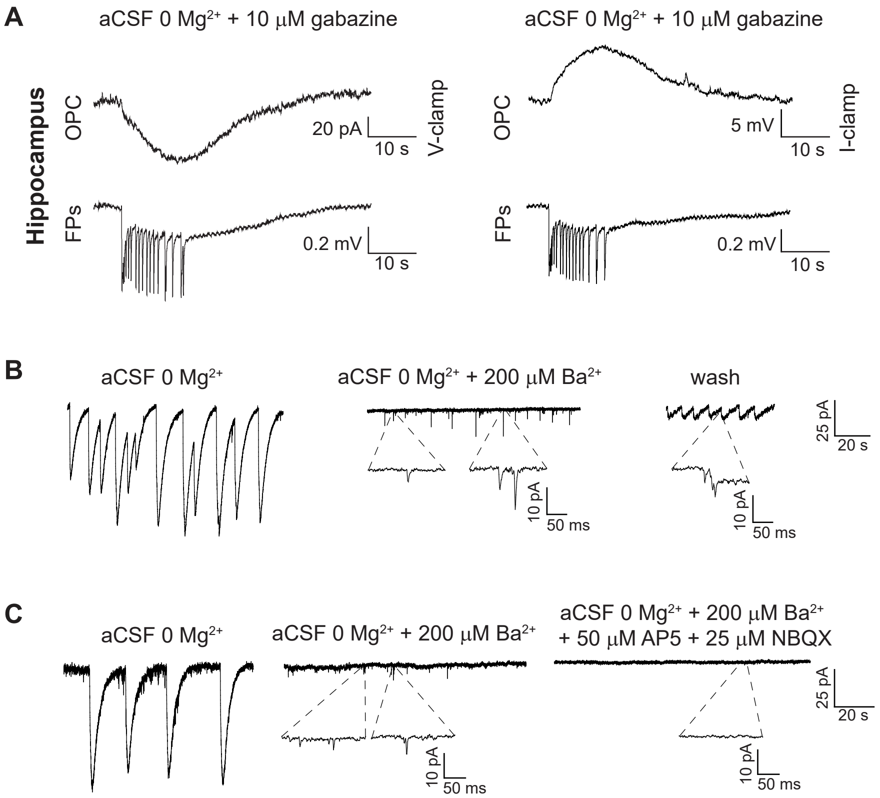

Figure S5. SLICs properties in hippocampus and in the cortex, related to Figure 4.

(**A**) Representative traces of an OPC recorded in the stratum radiatum of the hippocampus in voltage-clamp and current-clamp mode together with simultaneous recording of neuronal activity using extracellular field potentials (FPs). High neuronal activity was evoked by using 0 Mg^{2+}/gabazine (10 µM) aCSF. SLICs are accompanied by simultaneous depolarisations in OPCs.

(**B**) Ba^{2+} ions blocked SLICs completely, however, they did not affect spontaneous excitatory postsynaptic currents (EPSCs) in OPCs showing that neuronal activity and synaptic activity is preserved in these conditions. The effect of Ba^{2+} ions on SLICs was partially reversible.

(**C**) EPSCs in OPCs were completely blocked by 50 µM AP5 and 25 µM NBQX.

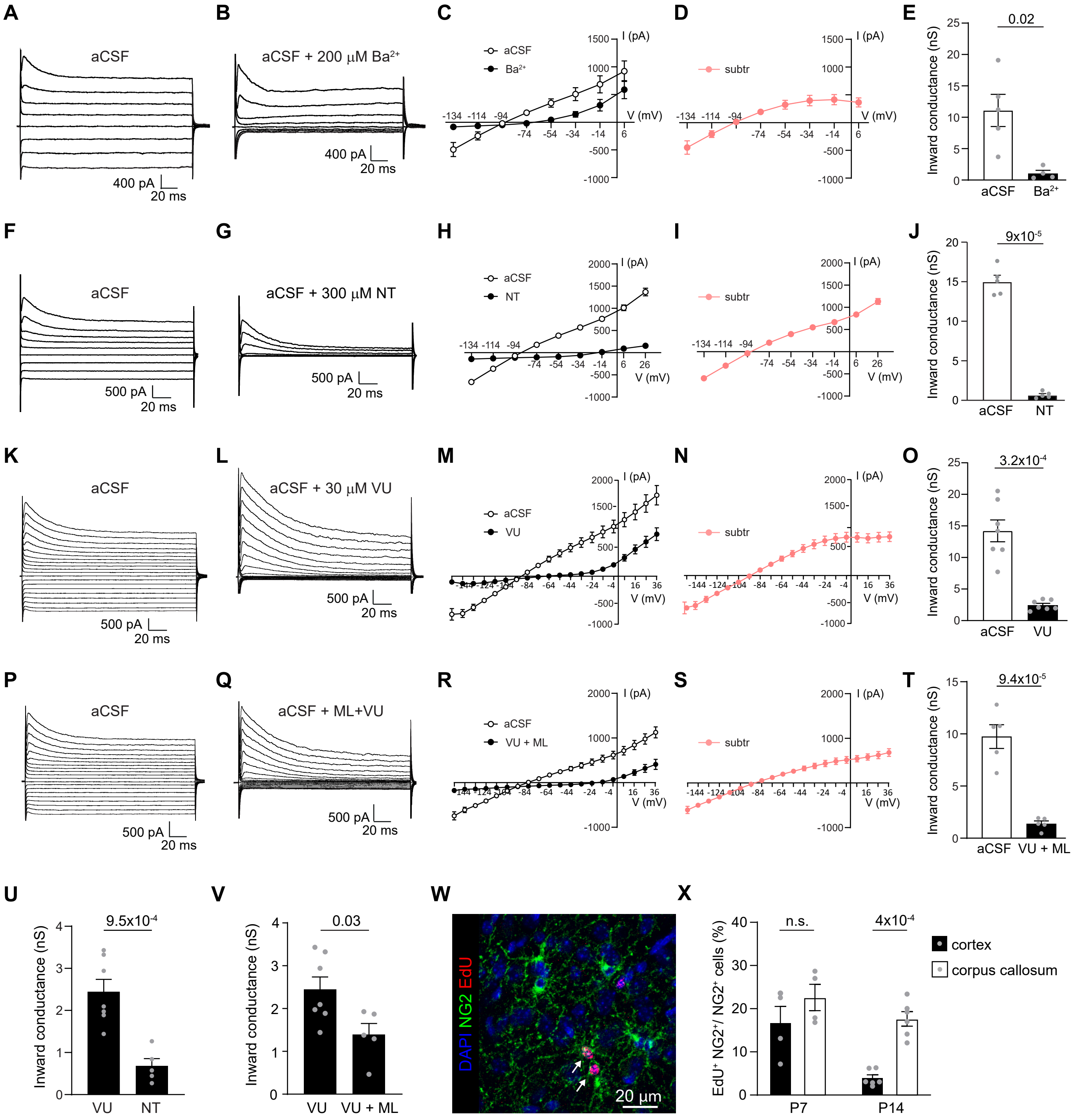

A B C D E
aCSF
aCSF + 200 µM Ba2+
400 pA 20 ms
aCSF Ba2+ subtr
I (pA) V (mV)
Inward conductance (nS)
0.02
F G H I J
aCSF + 300 µM NT
500 pA 20 ms
NT
9x10-5
K L M N O
aCSF + 30 µM VU
VU
3.2x10-4
P Q R S T
aCSF + ML+VU
VU + ML
9.4x10-5
U V W X
9.5x10-4
0.03
DAPI NG2 EdU
20 µm
EdU+ NG2+/ NG2+ cells (%)
n.s.
4x10-4
cortex
corpus callosum
P7 P14

Figure S6. Inward currents are mediated by K^+ conductance through Kir4.1 and Kir2.1 channels and correlate with OPC proliferation potential, related to Figure 4 and 6.

(**A,F,K,P**) Membrane currents evoked by stepping the membrane potential of recorded cells from -134 mV to +26 mV in 20 mV voltage steps (A,F), or from -154 mV to +36 mV in 10 mV voltage steps (K,P), in mouse cortical OPCs in control conditions.

(**B,G,L,Q**) Membrane currents evoked in OPCs after bath application of 200 μM Ba^{2+} (non-specific K^+ channel blocker), 300 μM nortriptyline (NT; inward rectifying K^+ channel blocker), 30 μM VU0134992 (a specific Kir4.1 channel blocker), and a combination of 30 μM VU0134992 + 30 μM ML133 hydrochloride (specific Kir4.1 and Kir2.1 channel blockers, respectively).

(**C,H,M,R**) Current/voltage (I/V) graphs of OPC membrane currents in control conditions and after application of different K^+ channel blockers.

(**D,I,N,S**) Current/voltage (I/V) graphs of the subtracted currents between the control conditions and after application of different K^+ channel blockers.

(**E,J,O,T**) Inward conductance in OPCs is blocked by different K^+ channel blockers.

(**U and V**) 30 μM VU alone does not block the inward K^+ currents completely, but 300 μM nortriptyline or the combination of 30 μM VU0134992 + 30 μM ML133 hydrochloride do block the currents entirely.

(W) Representative image of NG2 and EdU labelling in the cortex of a P14 mouse. White arrows indicate $NG2^+EdU^+$ cells.

(X) Mice were injected with 25 mg/kg EdU at P6 or P13 and perfused-fixed 24 hours later, at P7 or P14. At P7, before the onset of high inward conductance in the cortex, OPCs in the cortex and corpus callosum proliferate at similar rates. At P14, after the onset of high inward conductance in cortical OPCs, proliferation is reduced in cortical OPCs compared to callosal OPCs.

Dots represent individual recorded cells (E, J, O, T, U, V) or animals (X). p values are from two-tailed unpaired t-test or t-test with Welch's correction (E, J, O, T, U, V) or from two-way ANOVA with Šídák's multiple comparisons test (X). Data are presented as mean ± s.e.m..

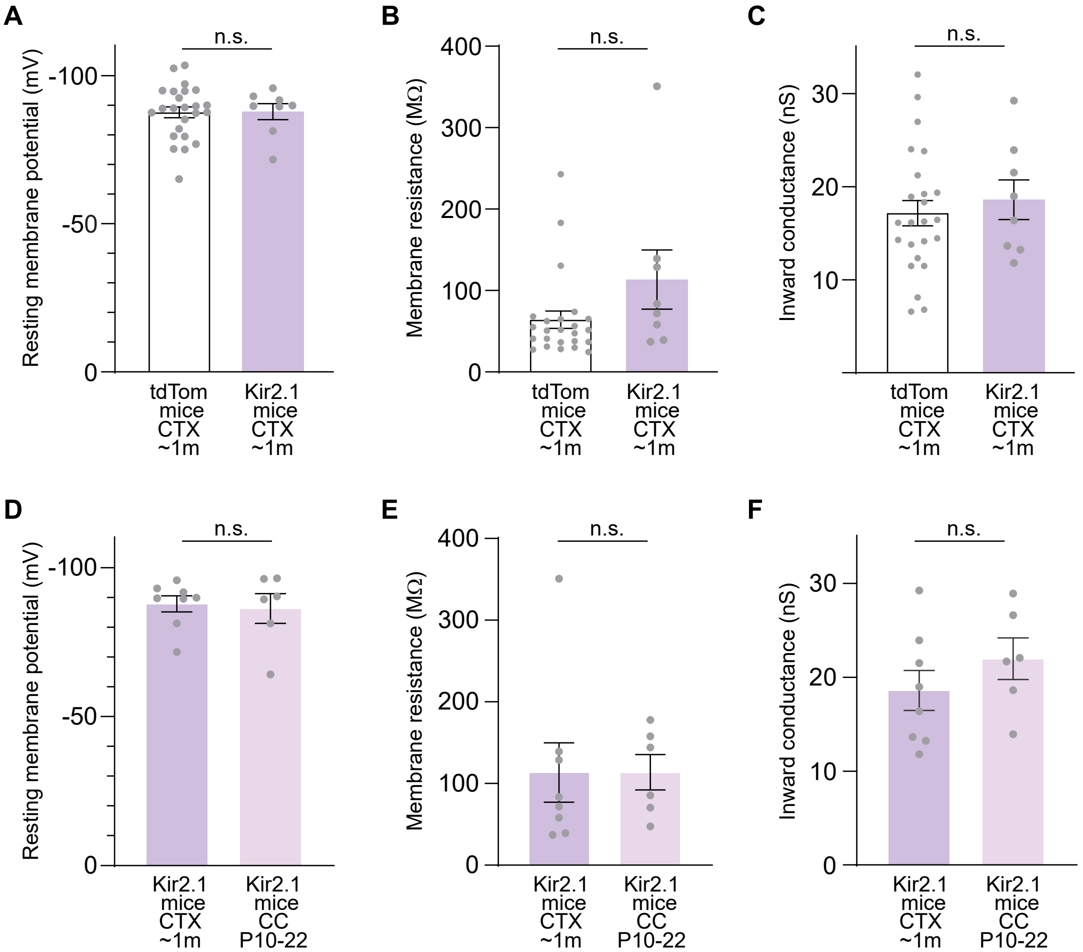

Figure S7. OPCs in Kir2.1 mice have physiological membrane properties and lose regional differences, related to Figure 6.

(**A**) The resting membrane potential, (**B**) membrane resistance, (**C**) and inward conductance in cortical (P25-30) OPCs overexpressing Kir2.1 channels are comparable to these passive membrane properties in age-matched wild type mice.

(**D**) The resting membrane potential, (**E**) membrane resistance, (**F**) and inward conductance in cortical and callosal OPCs overexpressing Kir2.1 channels in young animals are comparable.

Dots represent individual recorded cells. p values are from two-tailed unpaired t-test or t-test with Welch's correction. Data are presented as mean ± s.e.m..
