# Supplementary material for: Heterogeneity in oligodendrocyte precursor cell proliferation is dynamic and driven by passive bioelectrical properties
Source: Cell Rep. 2024 Oct 17;43(11):114873. doi: 10.1016/j.celrep.2024.114873 (PMC11602547; doi:10.1016/j.celrep.2024.114873)
Supplement: Document S2. Article plus supplemental information [file mmc2.pdf]

Cell Reports

Article

Heterogeneity in oligodendrocyte precursor cell proliferation is dynamic and driven by passive bioelectrical properties

Graphical abstract

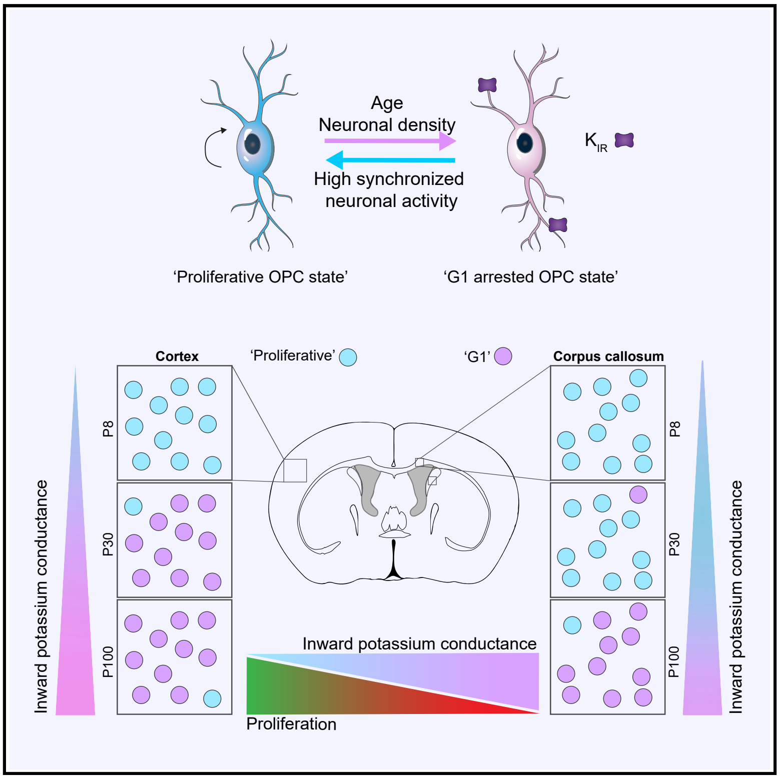

Highlights

- OPCs acquire inward potassium conductance with age
- Inward potassium conductance underlies OPC heterogeneity
- Inward potassium conductance inversely correlates with OPC proliferation
- Neuronal activity lowers inward potassium conductance and increases proliferation

Authors

Helena Pivoňková, Sergey Sitnikov, Yasmine Kamen, ..., Omar de Faria, Jr., Balazs Viktor Varga, Ragnhildur Thóra Káradóttir

Correspondence

rk385@cam.ac.uk

In brief

Pivoňková et al. show that oligodendrocyte precursor cells (OPCs) acquire inward potassium conductance at different times, causing regional heterogeneity, which, with age, becomes homogeneous again. Increasing inward conductance reduces OPC proliferation, keeping OPCs in cell-cycle arrest, while synchronized neuronal activity reduces inward potassium conductance, lifting the brake on proliferation.

Pivoňková et al., 2024, Cell Reports *43*, 114873
November 26, 2024 © 2024 The Author(s). Published by Elsevier Inc.
https://doi.org/10.1016/j.celrep.2024.114873

CellPress

Cell Reports

CellPress
OPEN ACCESS

Article

Heterogeneity in oligodendrocyte precursor cell proliferation is dynamic and driven by passive bioelectrical properties

Helena Pivoňková,[1,3,4] Sergey Sitnikov,[1,4] Yasmine Kamen,[1,4] An Vanhaesebrouck,[1] Moritz Matthey,[1] Sonia Olivia Spitzer,[1] Yan Ting Ng (吳胤霆),[1] Chenyue Tao (陶辰玥),[1] Omar de Faria, Jr.,[1] Balazs Viktor Varga,[1] and Ragnhildur Thóra Káradóttir[1,2,5,*]
[1]Cambridge Stem Cell Institute and Department of Veterinary Medicine, University of Cambridge, Cambridge CB2 0AW, UK
[2]Department of Physiology, BioMedical Center, Faculty of Medicine, University of Iceland, 101 Reykjavík, Iceland
[3]Present address: Department of Physiology, Second Faculty of Medicine, Charles University, 15006 Prague, Czechia
[4]These authors contributed equally
[5]Lead contact
*Correspondence: rk385@cam.ac.uk
https://doi.org/10.1016/j.celrep.2024.114873

SUMMARY

Oligodendrocyte precursor cells (OPCs) generate myelinating oligodendrocytes and are the main proliferative cells in the adult central nervous system. OPCs are a heterogeneous population, with proliferation and differentiation capacity varying with brain region and age. We demonstrate that during early postnatal maturation, cortical, but not callosal, OPCs begin to show altered passive bioelectrical properties, particularly increased inward potassium (K^+) conductance, which correlates with G1 cell cycle stage and affects their proliferation potential. Neuronal activity-evoked transient K^+ currents in OPCs with high inward K^+ conductance potentially release OPCs from cell cycle arrest. Eventually, OPCs in all regions acquire high inward K^+ conductance, the magnitude of which may underlie differences in OPC proliferation between regions, with cells being pushed into a dormant state as they acquire high inward K^+ conductance and released from dormancy by synchronous neuronal activity. Age-related accumulation of OPCs with high inward K^+ conductance might contribute to differentiation failure.

INTRODUCTION

Myelination is a protracted process whereby oligodendrocyte precursor cells (OPCs) differentiate into myelin-forming oligodendrocytes. Myelin is essential for fast signal transmission, synchronization of neuronal signals,[1] and metabolic support to axons.[2] Growing evidence shows that myelin plays a role in learning and memory and that neuronal activity is a regulatory signal for myelination and remyelination in young adults.[1,3–7] Learning and memory, myelin maintenance, and regeneration decline with age,[3,8–11] and white matter lesions accumulate.[12] Conceivably, this is in part due to reduced potential for OPCs to proliferate, differentiate, and maintain and regenerate myelin.[3,11]

Unlike other neural precursors, OPCs remain as the main proliferative cell in the adult brain, evenly distributed throughout the central nervous system (CNS).[13] This abundance and distribution have driven the presumption that OPCs have other functions beyond differentiating into oligodendrocytes. This is reinforced by accumulating evidence that OPCs are heterogeneous. OPCs respond differently to growth factors[14] and cytokines[15] and have different transcriptional profiles.[16] Depending on the region, OPCs differ in their capability to proliferate and differentiate into myelinating oligodendrocytes.[17–20] Active bioelectrical properties (e.g., voltage-gated ion channels and neurotransmitter receptor densities) reportedly differ between gray and white matter OPCs,[21,22] although this is disputed.[23,24] These contrasting findings may be explained by differences in the ages observed, as OPCs begin as a homogeneous population that becomes heterogeneous with age and region, reflecting differences in their capability to sense and respond to neuronal activity.[21] However, it is not yet clear whether this heterogeneity indicates different dynamic cell states[25] or terminal maturation into distinct functional OPC subgroups,[19] akin to the development of discrete GABAergic interneuron subtypes.[26]

One critical mechanism of neuronal plasticity is modulation of passive bioelectrical properties of neurons (i.e., potential, resistance, and passive conductance [e.g., inward potassium (K^+) conductance] of the cell membrane at rest). Changes in passive bioelectrical properties regulate how neurons sense or respond to incoming signals by modifying the driving force of ions across ionotropic receptors, adjusting the likelihood of summation of inputs, and altering the action potential threshold. The main features of passive bioelectrical properties are driven by a passive membrane K^+ conductance,[27–29] mainly through inward rectifying K^+ (Kir) channels. Although Kir channels in mature

© 2024 The Author(s). Published by Elsevier Inc.
This is an open access article under the CC BY license (http://creativecommons.org/licenses/by/4.0/).

oligodendrocytes are well described,[30] their function in OPCs is more enigmatic. A distinct feature of some adult OPCs in the CNS gray matter is a large passive inward K^+ conductance,[27–29] thought to be mediated by Kir channels, such as Kir4.1, Kir5.1, and Kir2.1.[31–35] Yet, oligodendrocyte lineage-specific Kir4.1-knockout studies have failed to detect any major differences in myelination,[32,36] although regional differences in OPC proliferation rate seem to be diminished.[37] It has been proposed that OPCs in the adult brain, particularly those with high inward K^+ conductance, have a function other than being a precursor to myelinating oligodendrocytes.[28] Reinforcing this idea, adult gray matter OPCs can modulate neuronal network activity by altering neuronal synaptic function,[38] and the observed increase in inward K^+ conductance in adult OPCs affects neuron-OPC communication.[28] OPCs in the mouse gray matter[20,28] and in high-density neuronal areas in the zebrafish[19] differ in proliferation and differentiation capacities from OPCs in the white matter or low-density neuronal areas. Cumulatively, these observations have supported the conclusion that adult OPCs may have become functionally different cell types.

An alternative conclusion may be that gray matter OPCs are in a different cell state, as ion channel expression differs throughout the cell cycle.[21,25,39] Importantly, cell cycle progression is, for example, regulated by different K^+ channels. For instance, voltage-gated K^+ channels promote proliferation,[39] and this seems to be the case also for OPCs,[40,41] whereas Kir channels regulate the length of G1 phase or the timing of G1/S phase transition[39]; however, their role in OPCs is unclear.

We therefore asked whether passive bioelectrical properties mediated by inward K^+ conductance mark a distinct subtype of OPCs or an adaptable cell state, given that increased K^+ conductance is known to regulate cell cycle time and cell fate.[39] We used single-cell electrophysiological recordings in heterozygous NG2-EYFP knockin mice, allowing for unbiased sampling of this population. We report that OPCs start out as a homogeneous population, but develop differences in passive bioelectrical properties, such as inward K^+ conductance, varying both within and between brain regions and with age. Neuronal activity and the local environment around individual OPCs seem to bidirectionally modulate their inward K^+ conductance. Conversely, inward K^+ conductance in OPCs determines their sensitivity to neuronal activity and inversely correlates with their proliferation potential by keeping them in the G1 stage of the cell cycle.

RESULTS

Passive bioelectrical properties of cortical, but not callosal, OPCs diverge during early CNS maturation

To investigate passive bioelectrical properties (i.e., resting membrane potential [RMP], membrane resistance [Rm], and passive membrane conductance [e.g., inward K^+ conductance]) of OPCs from development to middle age, we voltage-clamped OPCs in the cortex (CTX; Figure 1A) and corpus callosum (CC; Figure 1C) in NG2-EYFP-knockin heterozygous mice (NG2-EYFP). In these mice, all parenchymal EYFP-positive cells are Olig2 and NG2 positive throughout life, and EYFP expression tightly follows the expression of the NG2 protein.[42] Passive bioelectrical properties are indistinguishable between OPCs in white and gray matter during the first postnatal week, prior to the onset of myelination in the corpus callosum, like other OPCs' bioelectrical properties[21] (Figures 1B–1D, 1E, and S1, left column). However, after postnatal day (P) 9, when myelination begins in the corpus callosum, the passive bioelectrical properties begin to steeply change in cortical but not callosal OPCs (Figures 1B–1D and 1F). Accordingly, RMP, Rm, and passive membrane conductance (determined as the slope of the inward current; see STAR Methods) change steadily during the first month of life in cortical OPCs (Figures 1J–1L), whereas they remain stable in callosal OPCs (Figures 1M–1O). These passive bioelectrical properties diverge at a similar time compared to other bioelectrical properties of OPCs.[21] The passive bioelectrical properties of OPCs in the cortex and corpus callosum remain different for the first few weeks of life, but when myelination in the corpus callosum reaches steady state,[43,44] callosal OPCs begin to exhibit an increase in inward conductance and corresponding changes in RMP and Rm (Figures 1M–1O). By P100, the inward conductance in callosal OPCs becomes more like that of OPCs in the cortex (Figures 1I and S1, right column). After this age-dependent shift, the inward conductance remains relatively stable. These data show that gray matter OPCs become more hyperpolarized prior to the onset of myelination, with lower Rm and higher inward conductance, compared to white matter OPCs. However, this hyperpolarized state is not a unique feature of cortical OPCs, as subventricular zone (Figure S2) and corpus callosum OPCs enter this stage eventually; callosal OPCs do so when the myelination rate reaches a steady state. The timing of the onset of high inward conductance could in part explain the reduced proliferation and differentiation potential of cortical OPCs in young adults, and aged OPCs in general, given that passive bioelectrical properties modulate how cells respond to different environmental signals.

OPCs with high inward conductance reside in areas with high neuronal density

While the overall picture is that cortical OPCs diverge from callosal OPCs during early development, a more detailed analysis of the variance of inward conductance reveals that cortical OPCs are not homogeneous (Figures 1L, S3A, and S3B). The variance of inward conductance recorded in cortical OPCs increases sharply after P9 and by the end of the first month remains relatively constant at higher levels (Figure S3A). To establish whether this change in variance implies that there is more than one population of cells in the cortex, we plotted a population curve of the inward conductance and applied a Gaussian fit, where the sum of two Gaussians fits the data better than a single Gaussian ($p = 5.3 \times 10^{-5}$; Figure 2A). This reveals two clear populations of OPCs, one with low and one with high inward conductance. At the 90th percentile of the Gaussian fit of both Gaussian curves they merge to an equal conductance (2.35 nS), a value that divides the two populations, one with high and one with low inward conductance (Figures 2A and S3B). The relative distribution of OPCs with high inward conductance increases with age in both the cortex and the corpus callosum, although their proportions differ between regions (Figures S3C and S3D).

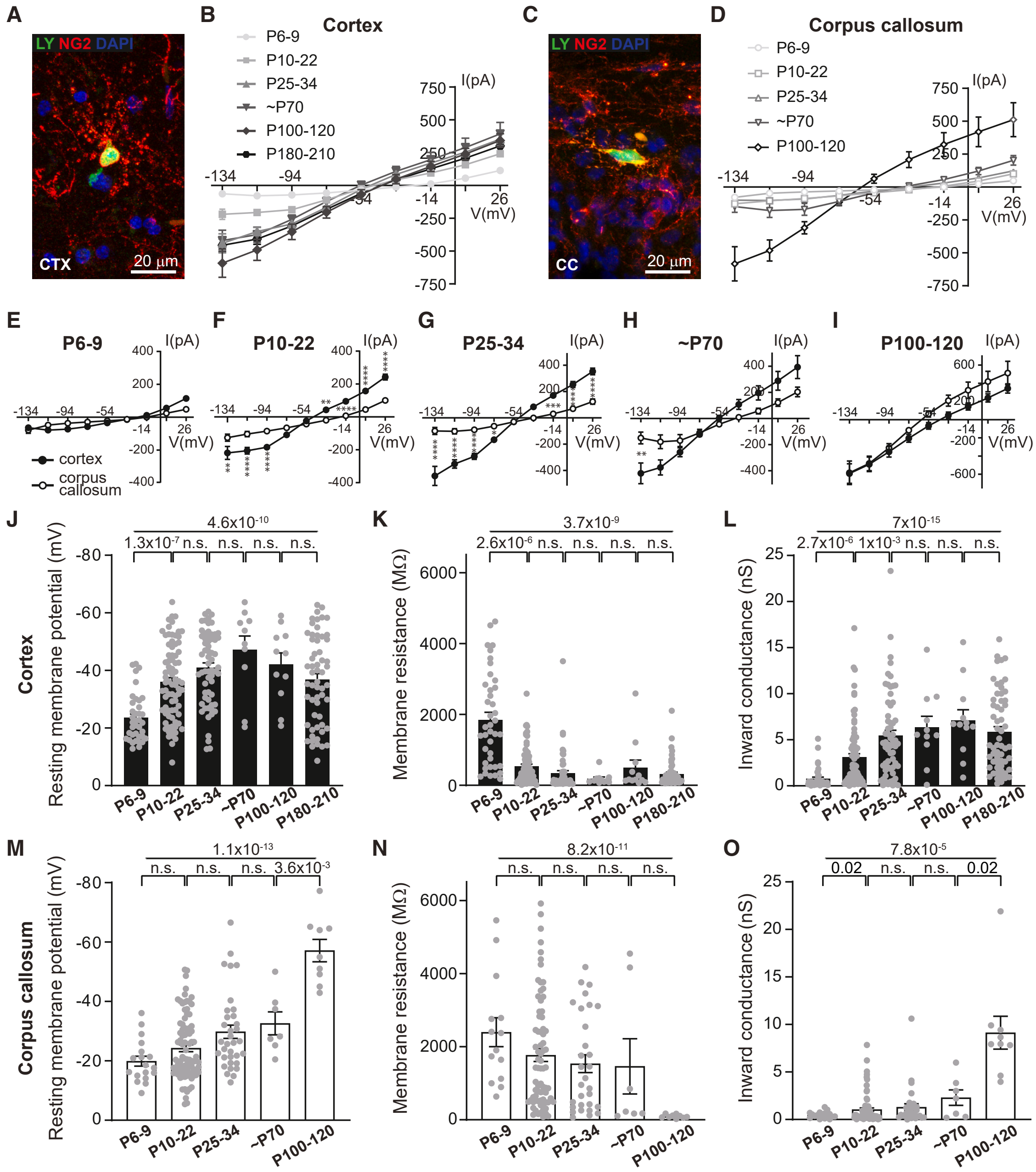

Figure 1. Cortical, but not callosal, OPCs develop inward conductance during early CNS maturation

(A and C) Cells were selected by their EYFP expression (NG2-EYFP-knockin mice) in the cortex (CTX; A) and corpus callosum (CC; C). During whole-cell patch-clamp recording, OPCs were dye-filled with Lucifer yellow (LY; green) and *post hoc* labeled for NG2 (red).

(B and D) Current/voltage (I/V) curves of steady-state currents in response to 20 mV steps from −134 to +26 mV in OPCs between P6 and P210 in CTX (B) and CC (D).

(E–I) Comparison of I/V curves between CTX and CC OPCs at different ages.

(J–L) In cortical OPCs, the resting membrane potential, membrane resistance, and inward conductance (see STAR Methods) change significantly at P10–P22 compared to P6–P9. These parameters then stay constant until P180–P210 except for the inward conductance, which further increases at P25–P34.

(M–O) In callosal OPCs, the resting membrane potential, membrane resistance, and inward conductance change substantially only at P100–P120.

Dots on bar graphs represent individual recorded cells. The *p* values are from two-way ANOVA with Šídák's multiple comparisons test (E–I), with $*p < 0.05$, $**p < 0.01$, $***p < 0.001$, and $****p < 0.0001$, or from one-way ANOVA or Welch's ANOVA (J–O; top) with *post hoc* Holm-Bonferroni tests (J–O; bottom). Data are shown as the mean ± SEM. See also Figure S1.

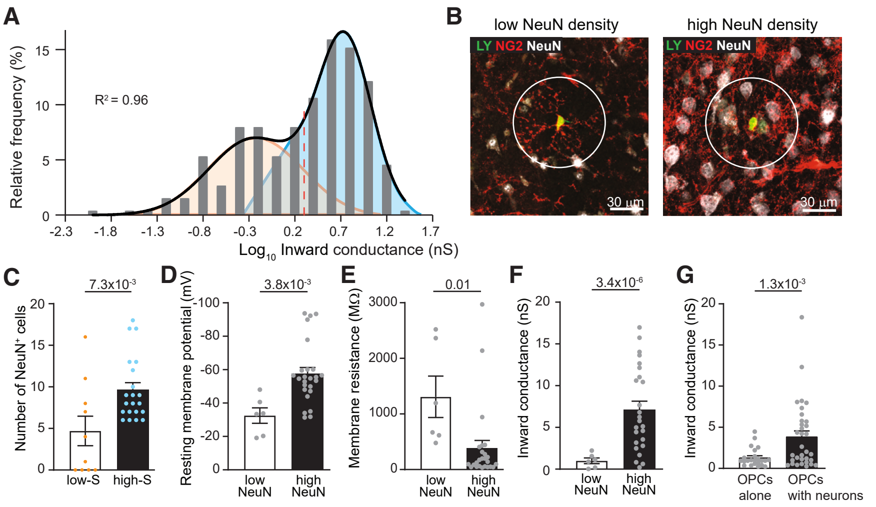

Figure 2. Cortical OPCs have different properties in areas of high neuronal density

(A) Gaussian fit of the log_{10}-transformed inward conductance (nS) in cortical OPCs ($n = 264$), where the sum of two Gaussians, R^2 value of 0.96, fits the data better than a single Gaussian. The red dashed line indicates the 90th percentile of the two Gaussian curves (equal to 2.35 nS).

(B) Delineation (white circle) of an area occupied by a recorded NG2$^+$ OPC (red) and dye filled with LY (green). Neuronal somata were labeled with anti-NeuN antibody (white).

(C) The number of NeuN$^+$ cells, or neuronal density, around OPCs with high inward conductance (high S; blue dots) was significantly higher compared to OPCs with a low inward conductance (low S; orange dots).

(D and E) OPCs surrounded by a high number of NeuN$^+$ cells were significantly more hyperpolarized (D) and had lower membrane resistance (E) compared to OPCs with a low number of NeuN$^+$ cells in their surroundings.

(F) The inward conductance was higher in OPCs with a high number of NeuN$^+$ cells around compared to OPCs with a low number of NeuN$^+$ cells in their surroundings.

(G) The inward conductance was higher in OPCs co-cultured with a high density of neurons compared to OPCs cultured alone.

Orange, blue, and gray dots represent individual recorded cells. The *p* values for (C)–(G) are from unpaired two-tailed t tests or t tests with Welch's correction. Data are shown as the mean ± SEM. See also Figure S3.

Zebrafish studies have indicated that OPC physiology differs between gray matter areas of high neuronal density and of low neuronal density but with high axonal density.[19] Thus, we investigated the cellular environment of recorded cortical OPCs by dye-filling them via the recording pipette and quantifying the density of neurons (NeuN$^+$ somata) around them. We marked the area occupied by an OPC by enlarging a circle from the soma to the extremities of its processes and then quantified the number of NeuN$^+$ somata and blood vessels within that area. The average circle diameter of a cortical OPC was 80 ± 2.7 μm, similar to previous findings[45] (Figure 2B). We found that OPCs with low inward conductance, resembling early postnatal OPCs, were on average in a lower neuronal density area within the cortex compared to OPCs with high inward conductance (Figure 2C), whereas the density of blood vessels did not correlate with high or low inward conductance ($p = 0.86$; low inward conductance, 0.7 ± 0.15 blood vessels; high inward conductance, 0.7 ± 0.11 blood vessels; two-tailed unpaired t test). We further investigated whether passive bioelectrical properties differed depending on whether cortical OPCs were in high or low neuronal density areas. OPCs in high neuronal density areas (defined from data in Figure 2C as having more than 5 NeuN$^+$ cells in their vicinity) have hyperpolarized RMP (Figure 2D), a larger membrane capacitance ($p = 0.02$; low neuronal density, 26 ± 5.9 pF [$n = 6$]; high neuronal density, 42.1 ± 2.9 pF [$n = 25$]; two-tailed unpaired t test), lower Rm (Figure 2E), and higher inward conductance (Figure 2F) compared to OPCs in low neuronal density areas. To test whether this is a general phenotype of OPCs around a high density of neurons, we cultured OPCs with a high density of cortical neurons and found that they have a larger inward conductance than OPCs cultured alone (Figure 2G). OPCs in a low neuronal density area within the cortex have passive bioelectrical properties more similar to those of age-matched OPCs in the corpus callosum, which is devoid of NeuN$^+$ neuronal somata (Figures S3E, S3F, and S3G), or to OPCs cultured alone (Figures S3H and S3I). From this, it seems that a subgroup of cortical OPCs in high neuronal density areas acquires a higher inward conductance, which is known to affect excitability, amplitude, temporal resolution of synaptic input detection, and temporal window for summation of inputs.[46] Conceivably, these changes could alter the way this subgroup of OPCs senses and responds to neuronal activity.

Cortical OPCs with high inward conductance respond to neuronal activity with transient inward currents

To test if the changes observed in cortical OPC passive bioelectrical properties affect how they sense neuronal activity, we augmented neuronal activity in acute brain slices by omitting Mg^{2+} from the artificial cerebrospinal fluid (aCSF), as this causes an overall increase in spontaneous activity in cortical neurons.[47] We observed transient inward currents in a subset of cortical OPCs (Figure 3A). These spontaneous long inward currents (SLICs) have a low frequency (0.09 ± 0.02 Hz, $n = 15$) with an amplitude of 34.7 ± 4.8 pA ($n = 53$), and they evoke a small depolarization (2.53 ± 0.97 mV; $n = 4$). The frequency of these events mirrors synaptic input frequency detected in OPCs voltage-clamped in a similar environment,[21] but their amplitude and duration more closely resemble the K^+-driven inputs evoked by high-frequency neuronal stimulation previously detected in cortical OPCs in adult animals.[28] To determine whether SLICs in OPCs are dependent on neuronal activity, we blocked neuronal activity with 1 μM tetrodotoxin (TTX), which completely blocked the currents (Figures 3B and 4H). To further investigate

this relationship, we simultaneously current clamped cortical neurons in layer 2/3 or 5 and voltage clamped nearby OPCs (Figure 3C). When recording in 0 Mg^{2+} aCSF solution, we found that neurons fired spontaneous bursts of action potentials that synchronized with SLICs recorded in nearby voltage-clamped OPCs, indicating that SLICs are phase locked to the action potentials (Figure 3D). In simultaneous dual voltage-clamp recordings from neurons and paired OPCs, we found that the SLICs in OPCs synchronized with large synaptic events recorded in the neurons (Figure 3E). However, as with the synaptic-like inputs in OPCs, the inputs recorded in the neurons slightly preceded the SLICs detected in the OPCs (Figure 3E′). These large synaptic inputs resemble events that are evoked by multiple synchronized inputs, presumably due to increased activity in the slice. To determine whether SLICs in OPCs are present only during high neuronal activity, we lowered neuronal activity by increasing Mg^{2+} in the aCSF. The synchronized large synaptic inputs detected in neurons in low Mg^{2+} disappear in high Mg^{2+}, but smaller synaptic events remain, whereas SLICs in OPCs are abolished (Figure 3F). We further investigated whether SLICs are detected in all OPCs in the cortex. We predominantly detected SLICs in OPCs around a high density of neuronal somata (Figure 3G). OPCs in which SLICs were detected had more negative RMP, lower Rm, and high inward conductance (Figures 3H–3K). These results indicate that SLICs are detected in a subset of cortical OPCs and are evoked by synchronized neuronal firing, presumably due to an increased K^+ release accompanying large, synchronized network events.

SLIC amplitude is dependent on the inward K^+ conductance in OPCs

To further investigate the relationship between SLICs, neuronal activity, and activity-induced changes in extracellular K^+ concentration ($[K^+]_e$), we simultaneously recorded OPCs in voltage-clamp mode together with extracellular field potentials (FPs), to assess neuronal activity, and K^+-ion-selective microelectrodes (K^+-ISMs), to record changes in $[K^+]_e$. The electrode used for FP recording and the K^+-ISMs were placed 50–100 μm deep into the acute brain slice, with under 10 μm difference in distance. OPCs were whole-cell patched within a 200 μm radius including the field and K^+-ISMs (the distance of individual OPC somata from the K^+-ISMs was 30–180 μm), and all three electrodes were always placed in the same cortical layer, either 2/3 or 5 (Figure 4A). Neuronal activity recorded as FPs slightly preceded the peaks of SLICs, which themselves coincided with the peaks of $[K^+]_e$ (Figure 4B). We next sought to determine the relationship between the SLIC amplitude and the $[K^+]_e$. K^+-ISM measurements of $[K^+]_e$ reflect the density of active neurons and the intensity of firing within the measured area,[48] and neuronal activity recorded in disinhibited conditions is similar within a cortical layer.[47,49] Therefore, as all electrodes were placed in one layer and as the events detected by all the electrodes were synchronized, we assume that all three electrodes were measuring events related to the activity of the same neuronal population and that the amount of K^+ released around the patched OPCs and the K^+-ISMs was similar. We found that $[K^+]_e$ between 0.14 and 1.32 mM evoked detectable SLICs in cortical OPCs. However, the amplitude of SLICs in OPCs did not correlate with $[K^+]_e$ (Figure 4C) but with Rm and the inward conductance of individual OPCs (Figures 4D and S4A). This is in contrast to previous ideas of high-K^+-conducting cortical OPCs being accurate sensors of local $[K^+]_e$.[28,50] Thus, we tested whether OPCs' inward conductance follows $[K^+]_e$ according to the Nernst equation (see STAR Methods), which it predictably did (Figures S4B and S4C). This shows that OPCs are not excellent sensors of $[K^+]_e$ per se, as the SLICs do not correlate to $[K^+]_e$, but that each cell with low Rm and high inward K^+ conductance is affected by $[K^+]_e$ in a predictable manner determined by its Rm and inward conductance.

To test whether SLICs are specific to a subset of cortical OPCs or a generic property of OPCs around a high density of neuronal somata, we recorded from OPCs in another area with high neuronal density. OPCs in the hippocampal stratum radiatum had passive bioelectrical properties similar to those of cortical OPCs located around a high density of neurons (e.g., Rm: stratum radiatum 161.02 ± 29.23 MΩ, n = 11; cortical OPCs in a high neuronal density area 393.62 ± 157.85 MΩ, n = 22; p = 0.16, two-tailed unpaired t test with Welch's correction; inward conductance: stratum radiatum 10.07 ± 1.39 nS, n = 11; cortical OPCs in a high neuronal density area 7.67 ± 1.1 nS, n = 22; p = 0.19, two-tailed unpaired t test). To determine if stratum radiatum OPCs also sense neuronal activity via K^+ signaling, we used previously described protocols to pharmacologically increase neuronal activity in acute hippocampal slices (P18–P22 mice).[51] This increased neuronal activity and evoked SLICs in hippocampal OPCs with similar amplitude (23.93 ± 10.8 pA, n = 4, p = 0.55, two-tailed unpaired t test) and depolarization (3.47 ± 2.33 mV, n = 3, p = 0.94, two-tailed unpaired t test) compared to cortical OPCs. However, unlike cortical OPCs, SLICs and depolarizations in hippocampal OPCs were longer and had a slower temporal pattern (0.03 ± 0.006 Hz, n = 4; Figure S5A). Perhaps this contrast between areas results from the difference in firing patterns of hippocampal and cortical neurons. To test this, we simultaneously recorded from OPCs in voltage-clamp mode together with recording extracellular FPs to assess neuronal activity and found that SLICs in hippocampal OPCs followed neuronal firing but were temporally distinct from those in the cortex (Figures 4B and S5A). The SLICs in OPCs followed closely the temporal rises in $[K^+]_e$, which oscillates in the cortex with a faster form of K^+ release (Figure 4B) compared to the slower sustained release in the hippocampus (Figure S5A), reflecting the pattern of neuronal activity. These data suggest that individual OPCs respond to subtle changes in $[K^+]_e$ and that the actual sensitivity of an individual OPC to changes in $[K^+]_e$ is set by its passive bioelectrical properties, which are predominantly determined by the membrane density of Kir channels.

SLICs in OPCs are mediated by Kir channels

To assess if SLICs are mediated by K^+ channels, we simultaneously recorded OPCs in voltage-clamp mode, FPs to assess neuronal activity, and changes in $[K^+]_e$ in the presence of K^+ channel blockers. First, we tested whether 200 μM Ba^{2+}, a non-specific blocker of K^+ channels, affected SLICs without altering network activity or $[K^+]_e$ elevation around OPCs. Ba^{2+} at 200 μM reversibly inhibited SLICs in OPCs, but did not affect

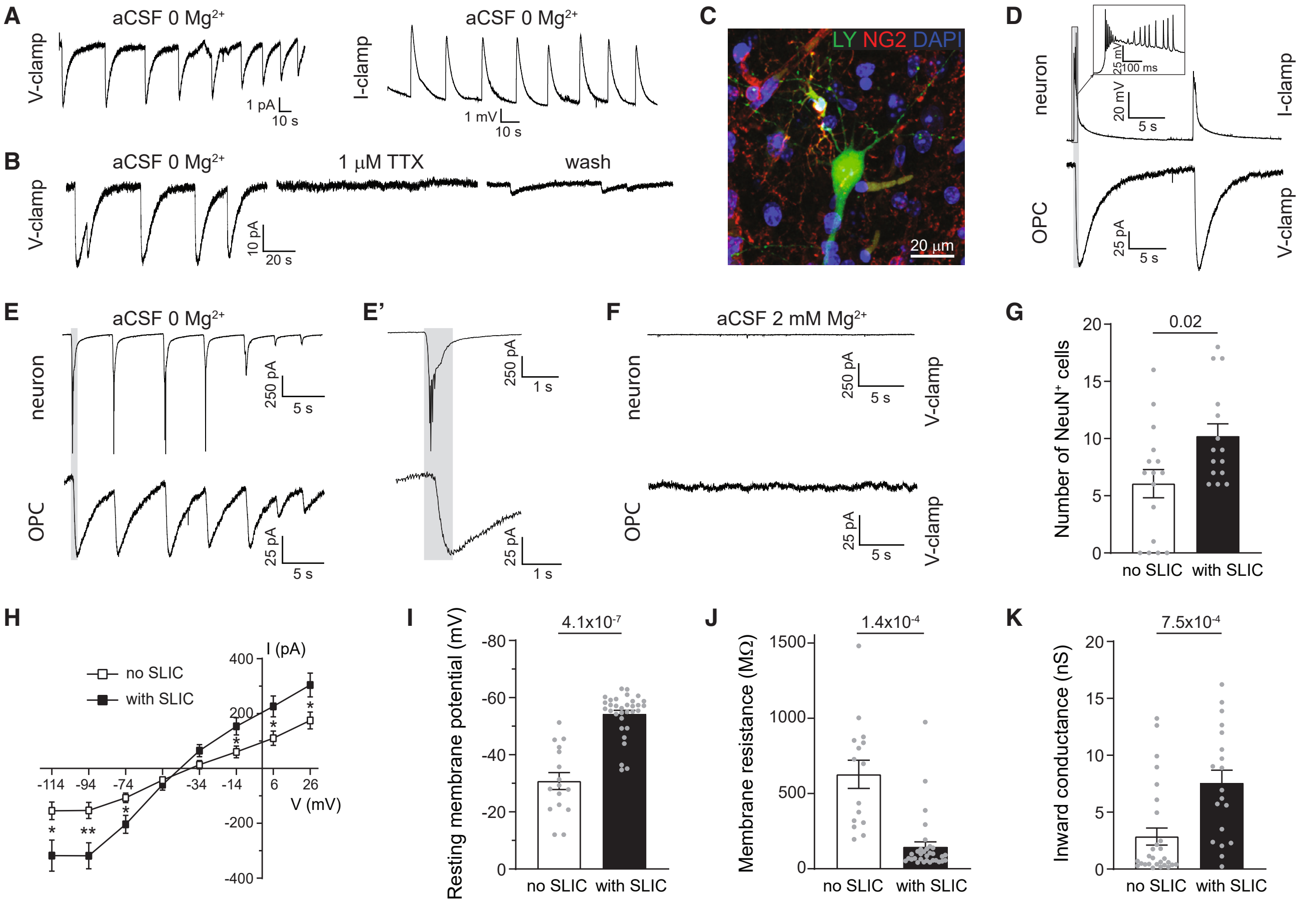

Figure 3. Neuronal activity induces spontaneous long inward currents in cortical OPCs

(A) Increasing neuronal firing by omitting Mg^{2+} in the aCSF evokes spontaneous long inward currents (SLICs) in OPCs, detected both in voltage-clamp and in current-clamp mode.

(B) SLICs are reversibly blocked by tetrodotoxin (TTX), suggesting that they are dependent on neuronal activity.

(C) OPCs and neurons were simultaneously patch clamped and dye-filled with Lucifer yellow (LY). OPCs were labeled for NG2 (red).

(D) Dual patch-clamp recordings from a neuron in current-clamp and an OPC in voltage-clamp mode. Neurons in disinhibited slices (0 Mg^{2+} aCSF) fire spontaneous action potentials, which slightly precede SLICs in OPCs, highlighted by a gray box.

(E and F) Dual voltage-clamp recordings from a neuron and an OPC show that adding 2 mM Mg^{2+} to the aCSF reduces the amplitude of synaptic inputs in neurons and the detection of SLICs in OPCs. Synaptic inputs to neurons slightly precede SLICs in OPCs, highlighted by a gray box and zoomed in (E′).

(G) OPCs showing SLICs have a higher number of $NeuN^+$ cells in their surroundings.

(H) OPCs with SLICs show higher inward conductance compared to OPCs without SLICs.

(I–K) OPCs with SLICs are more hyperpolarized and have lower membrane resistance and higher inward conductance than OPCs without SLICs.

Dots represent individual recorded cells. The p values are from two-way ANOVA with Šídák's multiple comparisons test (H), with $^*p < 0.05$ and $^{**}p < 0.01$, or unpaired two-tailed t tests or t tests with Welch's correction (G and I–K). Results are given as the mean ± SEM. See also Figures S4 and S5.

neuronal activity nor activity-dependent changes in $[K^+]_e$ (Figures 4E–4H). Furthermore, simultaneous voltage clamping of a neuron and a nearby OPC showed that 200 μM Ba^{2+} blocked SLICs in OPCs without affecting neuronal activity-dependent synaptic inputs (Figure 4F) or synaptic inputs from neurons to OPCs (Figure S5B), which were blocked by glutamate receptor antagonists (Figure S5C). These results show that Ba^{2+} does not block neuronal activity, nor does it affect $[K^+]_e$, but it blocks K^+ channels that mediate SLICs in OPCs and particularly affects inward K^+ conductance (Figures S6A–S6E). Next we tested nortriptyline, a generic Kir channel antagonist,[52] as previously Kir channels have been described as the main contributors to passive inward conductance in OPCs.[28] Nortriptyline (300 μM) blocked SLICs (Figures 4G and 4H) and the inward current detected in the subset of cortical OPCs with high inward conductance (Figures 4I and S6F–S6J). This indicates that age-related inward currents in cortical OPCs and SLICs are mediated by Kir channels. To determine which Kir channels, we first blocked the weakly rectifying channel Kir4.1, known to be expressed in oligodendrocyte-lineage cells,[32,34,36] with a specific Kir4.1 channel blocker, VU0134992 (VU).[53] However, a highly saturating dose of VU did not fully block SLICs nor the inward conductance in OPCs (Figures S6K–S6O, S6U, and S6V). Therefore, we tested whether the addition of ML133 hydrochloride (ML),[54] a specific

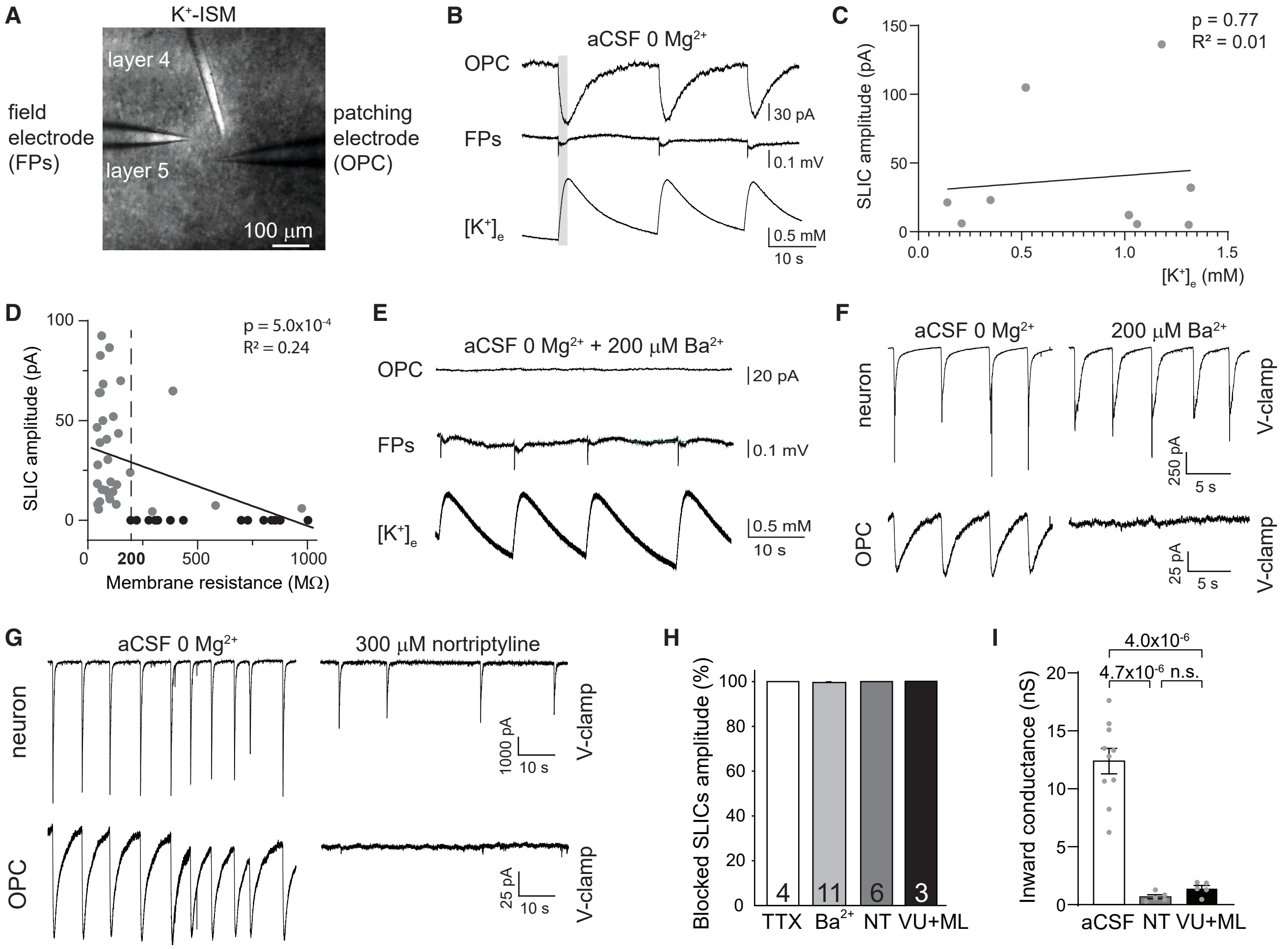

Figure 4. SLICs are mediated by inward rectifying K^+ channels

(A) Differential interference contrast image of the mouse cortex depicting the simultaneous triple recording from an OPC using patch clamp, extracellular field potentials (FPs) recording to assess neuronal activity, and K^+-selective microelectrodes (K^+-ISM) to record changes in extracellular K^+ concentration ($[K^+]_e$).

(B) Bath perfusion with 0 Mg^{2+} aCSF leads to spontaneous neuronal activity in mouse cortical slices as shown by FP recording accompanied by a release of K^+ ions from neurons into the extracellular space recorded by K^+-ISM and SLICs in OPCs. Representative traces are shown.

(C) The amplitude of SLICs does not correlate with $[K^+]_e$.

(D) SLICs occur mostly in OPCs with low membrane resistance.

(E and F) Adding Ba^{2+} blocks SLICs in OPCs but does not affect neuronal activity nor the release of K^+ ions from active neurons.

(G) Dual patch-clamp recordings from a neuron and an OPC in voltage-clamp mode showing that addition of nortriptyline blocks SLICs in OPCs but neuronal depolarizations are preserved.

(H) Quantification of the percentage of SLICs blocked by TTX, Ba^{2+} (non-specific K^+ channel blocker), nortriptyline (NT; inward rectifying K^+ channel blocker), and a combination of 30 µM VU0134992 (VU) + 30 µM ML133 (ML) hydrochloride (specific Kir4.1 and Kir2.1 channel blockers, respectively).

(I) Both nortriptyline and the combination of VU + ML block the inward currents in OPCs almost entirely (Welch's ANOVA, $p = 3.4 \times 10^{-6}$).

The numbers of recorded cells are indicated in the bars (H), while dots represent individual recorded cells (C, D, and I). The *p* values are from linear regression (C and D) or from Welch's ANOVA followed by Holm-Bonferroni *post hoc* tests (I). Data are presented as the mean ± SEM. See also Figure S6.

antagonist for the strong rectifying channel Kir2.1, would block the remainder of the current. A combination of VU and ML fully blocked both SLICs and inward conductance and consequently other passive bioelectrical properties to a similar level compared to nortriptyline (Figures 4H, 4I, and S6P–S6V). This indicates that age-related inward currents in cortical OPCs and SLICs are mediated by both Kir4.1 and Kir2.1 channels, linking the appearance of SLICs to the age-related increase in inward K^+ conductance in gray matter OPCs.

OPCs with high inward K^+ conductance rest in the G1/G0 phase of the cell cycle

We next sought to determine the functional role of SLICs in OPCs. We found that SLICs are detected only in OPCs with high inward K^+ conductance during periods of synchronized neuronal activity. They are reminiscent of spontaneous slow inward currents (SICs) recorded in proliferative glioma cells in the cortex, particularly during periods of high neuronal activity.[55,56] Indeed, neuronal activity has been shown to augment

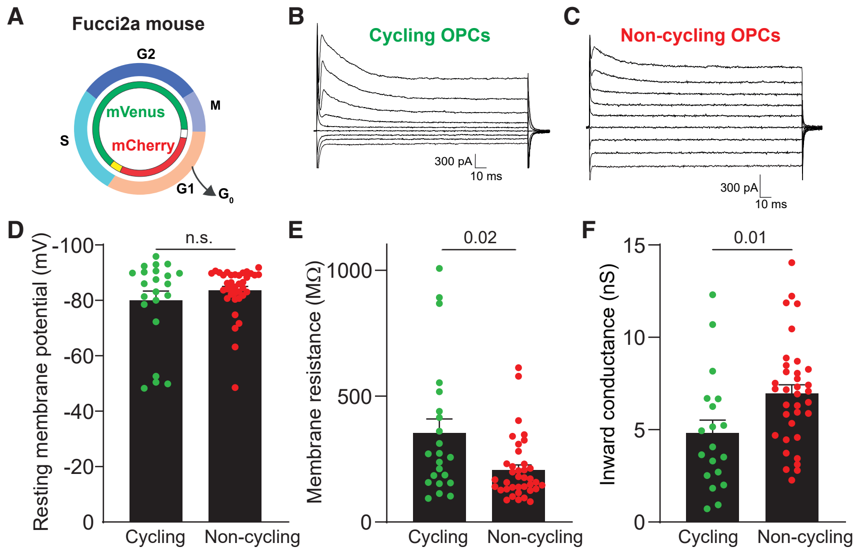

Figure 5. Cortical OPCs with high inward K⁺ conductance reside in the G1/G0 cell cycle phase

(A) Schematic showing the fluorescent labeling seen at different points in the cell cycle for fluorescent ubiquitination-based cell cycle indicator 2a (Fucci2a) mice.

(B and C) Membrane currents evoked by stepping the membrane potential of recorded cells from −134 to +26 mV in 20 mV voltage steps in the NG2-EYFP:Fucci2a mouse cortex either in green (cycling) or in red (non-cycling) OPCs.

(D) The resting membrane potential did not differ between the cycling (green) and the non-cycling (red) OPCs in the mouse cortex.

(E and F) The membrane resistance of non-cycling (red) OPCs was significantly lower (E) and the inward conductance (F) was significantly higher compared to cycling (green) OPCs.

Green or red dots indicate individual recorded cells. The *p* values were calculated by unpaired two-tailed t test. Data are shown as the mean ± SEM.

OPC proliferation.[6,7] Moreover, as cell proliferation, in general, seems to correlate with inward K^+ conductance,[39] we sought to test if high inward K^+ conductance is related to OPC proliferation. To assess the differences in inward K^+ conductance in cycling and non-cycling OPCs, we crossed NG2-EYFP mice with Fucci2a mice,[21] in which different phases of the cell cycle are distinguished by the expression of mCherry in the G1/G0 phase and mVenus in the G2/M phase (Figure 5A). We found that cortical OPCs (between P7 and P105) in the G1/G0 phase, non-cycling cells, had lower Rm and a larger inward conductance compared to cycling OPCs in the G2/M phase (Figures 5B–5F). These findings are consistent with proliferating cells having low inward K^+ conductance[57] and with the known role of membrane K^+ conductance in cycling cells.[39]

Increasing inward K⁺ conductance diminishes proliferation of OPCs

To determine the impact of increased inward K^+ conductance on OPC proliferation, we overexpressed Kir channels specifically in developing OPCs. The main Kir channels in OPCs are Kir4.1, Kir2.1, and Kir5.1,[21,35] where Kir5.1 is a non-functional channel. Our data show that both Kir4.1 and Kir2.1 mediate age-related changes in passive bioelectrical properties in OPCs, and these are also known to form functional heteromeric Kir4.1/2.1 channels in oligodendrocyte-lineage cells.[35,58] From our previous work, we find that gene expression for most Kir channels increases in OPCs with age, but the most prominent change is in Kir2.1,[21] which is a strong inward rectifier.[59] Now, we have shown that Kir2.1 functionally contributes to the age-related increase in inward K^+ conductance in OPCs (Figures 4I and S6P–S6V). Thus, to mimic the increase in inward conductance seen in OPCs with age, without affecting the voltage-gated outward conductance, we overexpressed Kir2.1 channels in OPCs by crossing PDGFRα-CreER^{T2} mice with STOP$^{fl/fl}$-Kir2.1-mCherry mice[60] (resulting offspring are referred to as Kir2.1 mice) or STOP$^{fl/fl}$-tdTomato mice[61] as controls. To initiate Cre-mediated recombination, tamoxifen was given to pups for 3 consecutive days and experiments were performed 3–10 days later. First, we voltage clamped Kir2.1-mCherry$^+$ or tdTomato$^+$ (control) OPCs in the corpus callosum, as at this age callosal OPCs do not have large endogenous inward K^+ currents (Figures 1E and 1O). OPCs overexpressing Kir2.1 channels were more hyperpolarized and had lower Rm and a higher inward K^+ conductance (Figures 6A–6C and 6E–6G), reminiscent of aged OPCs (Figures 1 and S1) and the subpopulation of OPCs located around a high density of neuronal somata and where SLICs are detected in the cortex of young adult animals (Figures 2, 3, and 4). At the age at which we detect increased inward K^+ conductance in cortical OPCs (Figures 1B and 1L), the passive bioelectrical properties of cortical OPCs overexpressing Kir2.1 were similar to same-age wild-type OPCs (Figures S7A–S7C). These data show that there is not an excessive overexpression and indicate that the Kir2.1 overexpression in early postnatal OPCs faithfully mimics the physiological changes in passive bioelectrical properties seen in aged OPCs and the subpopulation of OPCs around a high density of neurons. Similar changes in passive bioelectrical properties were detected when we overexpressed Kir4.1 channels in cultured OPCs (Figures 6D–6H) (RMP, control OPC cultures, −74.3 ± 1.6 mV; Kir4.1-transfected OPC cultures, −94.5 ± 0.5 mV; $p = 2.9 \times 10^{-15}$; Rm, control OPC cultures, 1,549.4 ± 479.1 MΩ; Kir4.1-transfected OPC cultures, 29.9 ± 5.3 MΩ; $p = 6.1 \times 10^{-5}$, two-tailed unpaired t test). To test the functional effect of increased inward K^+ conductance on proliferation, we gave a single 5-ethynyl-2’-deoxyuridine (EdU) injection to Kir2.1 mice at P13 (25 mg/kg intraperitoneally [i.p.]) and analyzed proliferation 24 h later. Both cortical and callosal NG2$^+$/Kir2.1-mCherry$^+$ cells proliferated less compared to NG2$^+$/mCherry$^-$ cells in Kir2.1 mice or NG2$^+$ cells in their wild-type littermates (Figures 6I and 6J). Likewise, overexpressing Kir4.1 channels in cultured OPCs reduced their proliferation capacity (Figure 6K). These data indicate that increasing inward K^+ conductance, as will eventually occur in OPCs with age, diminishes their capacity to proliferate.

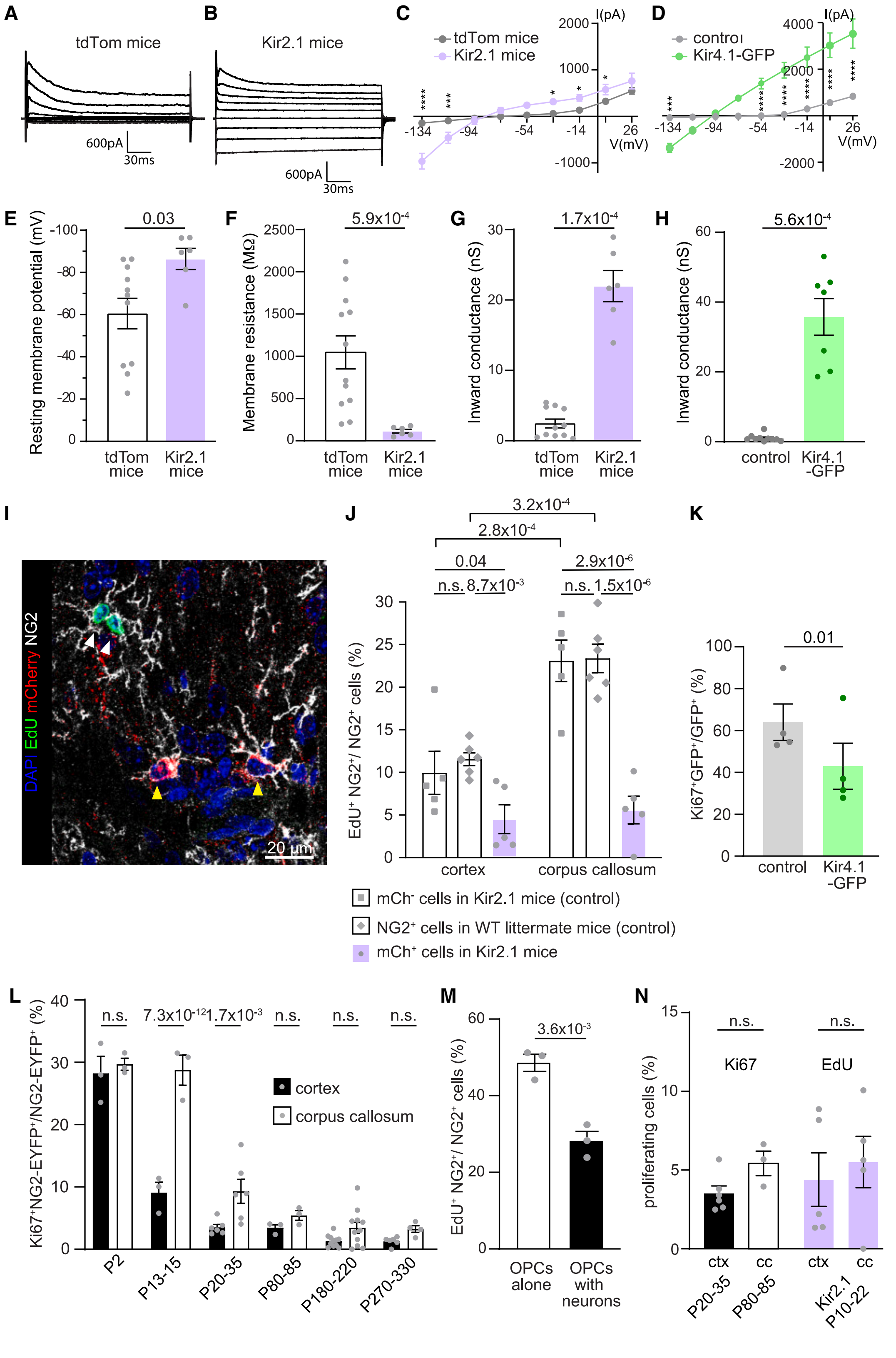

(legend on next page)

The time of onset of increased inward K^+ conductance differs between regions, potentially explaining the reported heterogeneity in OPC proliferation capacity between gray and white matter. To test this, we examined OPC proliferation at different postnatal ages in both gray and white matter. Before the onset of high inward K^+ conductance in the cortex, cortical and callosal OPCs proliferated at similar rates (Figures 6L, S6W, and S6X). At this time, both cortical and callosal OPCs lacked inward K^+ conductance and had indistinguishable passive bioelectrical properties. Once OPCs in the corpus callosum and cortex differed in inward K^+ conductance, there was a significant difference in their proliferation capacity (Figure 6L). Likewise, when we cultured OPCs with a high density of neurons, which increased inward K^+ conductance (Figure 2G), proliferation was significantly reduced compared to OPCs cultured alone (Figure 6M). Similarly, when callosal OPCs began to exhibit increased inward K^+ conductance and their passive bioelectrical properties again became near indistinguishable from those of cortical OPCs (after P70; Figure S1), their proliferation rates became similar (Figures 6L and 6N). When Kir2.1 channels were overexpressed in young OPCs, artificially making gray and white matter OPCs' passive bioelectrical properties similar (Figures S7D–S7F), the difference in proliferation between the corpus callosum and the cortex disappeared (Figure 6N). This suggests that the reported OPC heterogeneity between white and gray matter, or areas of high and low neuronal density, is due to temporal differences in their bioelectrical properties and not different fates and that signals that alter passive bioelectrical properties in OPCs modulate their proliferative capacity.

Stimulation of neuronal activity *in vivo* leads to a decrease in the inward K^+ conductance in OPCs and an increase in proliferation

We find that in the young adult cortex, a subset of OPCs have a high inward K^+ conductance and seem to be in the G1/G0 phase of the cell cycle, but at the same time are also capable of sensing changes in $[K^+]_e$ evoked by high neuronal firing. As increased neuronal activity in the young adult has been shown to increase cortical OPC proliferation and myelination,[7] we asked if increased inward K^+ conductance keeps OPCs in the G1/G0 phase until activity is sufficient to evoke SLICs. To increase neuronal activity *in vivo*, we expressed the hM3Dq DREADD (designer receptor exclusively activated by designer drugs) in neurons, which causes their depolarization in response to activation by the synthetic ligand clozapine-N-oxide (CNO)[62] or its metabolite clozapine.[63] We stereotaxically bilaterally injected adult NG2-EYFP mice in the lateral somatosensory cortex with pAAV8-hSyn-hM3D(Gq)-mCherry (Gq) or pAAV8-hSyn-mCherry (control) viruses and allowed at least 3 weeks for transgene expression (Figures 7A–7F and 7G). We administered CNO in the drinking water (25 mg/L) for 7–9 days to increase overall activity. This approach has been shown to increase neuronal activity by depolarizing the membrane potential in Gq-expressing neurons.[64] We found over 2.5-fold increase in cFos activation in animals transfected with Gq compared to controls following CNO administration (Figures 7B and 7C) and increased frequency of synchronous neuronal activity events in the cortex that evoke SLICs in the subset of OPCs with high inward K^+ conductance (Figure 4) during acute CNO administration on brain slices (Figures 7D and 7E). After 7–9 days of increased neuronal activity, OPCs were whole-cell patched in the cortical areas close to transfected neurons expressing either Gq or control virus identified by high mCherry protein expression (Figure 7G). We found that OPCs located in areas where we increased the firing rate of neurons had an RMP similar to that of controls (Figure 7H) but higher Rm (Figure 7I) and reduced inward K^+ conductance (Figure 7J). These data indicate that inward K^+ conductance in OPCs might be regulated by neuronal activity and that cortical OPCs reduce their inward K^+ conductance when exposed to increased neuronal activity. Importantly, these changes in neuronal activity and, subsequently, passive bioelectrical properties led to a clear increase in OPC

Figure 6. Increasing inward K^+ conductance in OPCs diminishes their proliferation

(A and B) Membrane currents evoked by stepping the membrane potential from −134 to +26 mV in 20 mV voltage steps in OPCs recorded in the corpus callosum of PDGFRα-CreER^{T2}:Kir2.1-mCherry mice or PDGFRα-CreER^{T2}:tdTomato reporter mice.

(C) I-V curve of steady-state currents in response to 20 mV steps between −134 and +36 mV in Kir2.1 and tdTom mice. Kir2.1 channel overexpression in OPCs increases predominantly inward K^+ currents.

(D) I-V curve of steady-state currents in response to 20 mV steps between −134 and +36 mV in Kir4.1 channel-overexpressing and control cultured OPCs. Kir4.1 channel overexpression in OPCs produces passive (ohmic) K^+ currents.

(E–G) mCherry$^+$ OPCs in Kir2.1 mice have significantly lower membrane potential, lower membrane resistance, and higher inward conductance compared to tdTom$^+$ control OPCs.

(H) Cultured OPCs overexpressing Kir4.1 channels have higher inward conductance compared to control cultured OPCs.

(I) Quantification of OPC proliferation in Kir2.1 mice and their WT littermates in brain slices stained with DAPI (blue), EdU (green), mCherry (red), and NG2 (white). mCherry$^+$/EdU$^-$ OPCs are indicated with yellow arrowheads and mCherry$^-$/EdU$^+$ OPCs are indicated with white arrowheads.

(J) Proliferation is reduced in Kir2.1-mCherry$^+$ OPCs compared to controls.

(K) Proliferation is reduced in Kir4.1 channel-overexpressing cultured OPCs compared to controls.

(L) Proliferating OPCs in NG2-EYFP mice were immunolabeled with Ki67 at different ages. At P2, before the onset of high inward conductance, OPCs in the cortex and corpus callosum proliferate at similar rates. After the onset of high inward conductance in cortical OPCs, callosal OPCs proliferate more compared to cortical OPCs. At P80, the proliferation rates in both areas become similarly low.

(M) OPCs co-cultured with neurons proliferate significantly less compared to OPCs cultured alone.

(N) The rate of proliferation is comparable between young cortical and adult callosal OPCs, as well as between P10–P22 cortical and callosal OPCs overexpressing Kir2.1 channels.

Individual dots indicate individual recorded cells (E–H), average in an animal (J, L, and N), or average on a coverslip (K and M). The number of animals was 5–6 per group. The p values are from two-way ANOVA with Šídák's multiple comparisons test (C and D), with $^*p < 0.05$, $^{**}p < 0.01$, $^{***}p < 0.001$, and $^{****}p < 0.0001$, unpaired two-tailed t tests (E–H, K, M, and N) or mixed-effects analysis (J and L). Results are given as the mean ± SEM. See also Figures S6 and S7.

CellPress
OPEN ACCESS

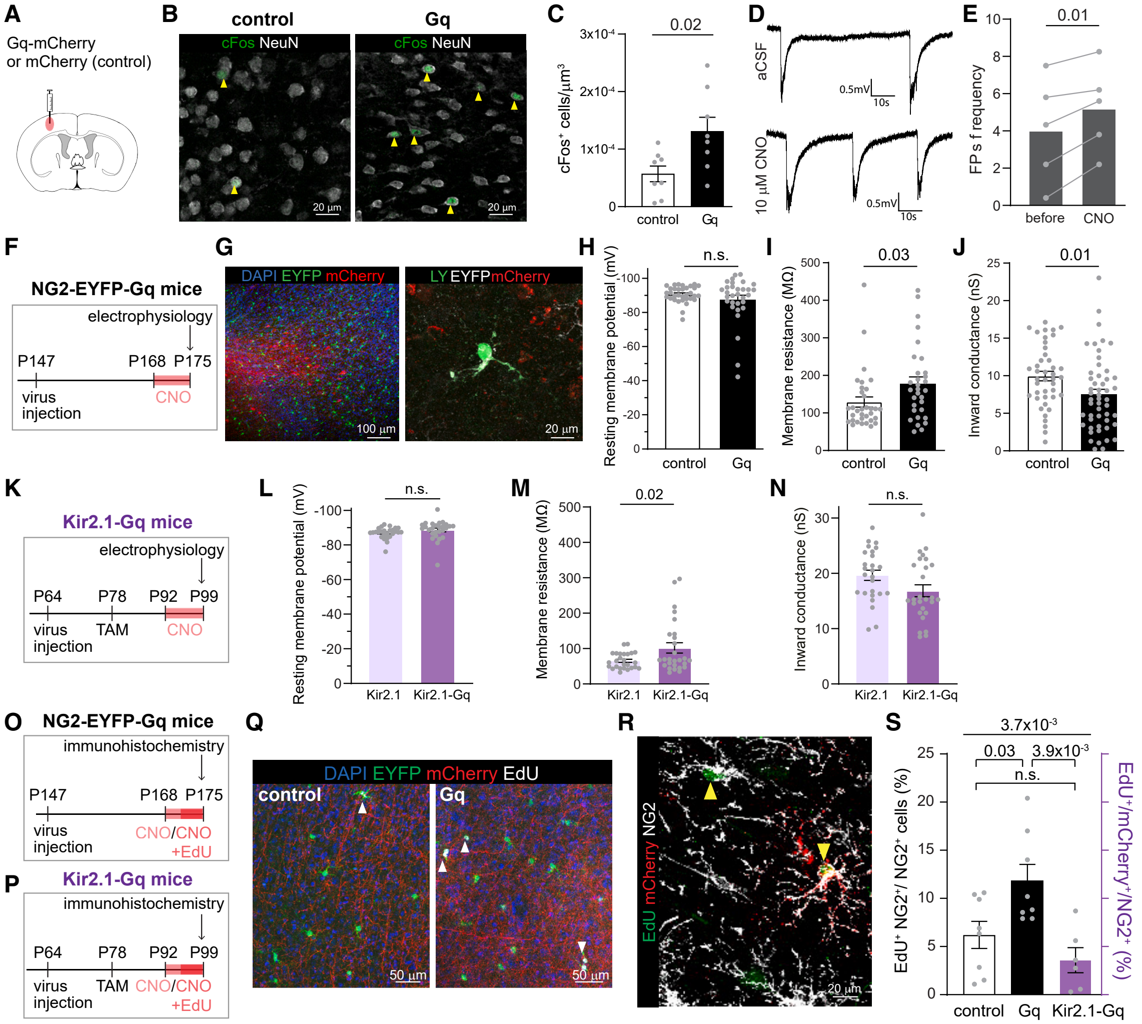

Figure 7. Increased neuronal activity leads to a decrease in inward K$^+$ conductance and an increase in proliferation in OPCs

(A) Adult NG2-EYFP mice were stereotaxically injected into the somatosensory cortices with either pAAV8-hSyn-hM3D(Gq)-mCherry (Gq) or pAAV8-hSyn-mCherry (control) viruses.

(B) Representative images of cFos (green) and NeuN (white) staining in control and Gq mice. Yellow arrowheads point to NeuN$^+$/cFos$^+$ cells.

(C) The number of cFos$^+$ cells was higher in Gq animals compared to controls.

(D) Representative traces from cortical extracellular field potential recording in acute brain slices from Gq mice superfused either with aCSF or with aCSF containing 10 μM CNO.

(E) The frequency of neuronal firing was increased by 10 μM CNO in brain slices from Gq mice.

(F) Timeline of the experiment.

(G) EYFP$^+$ OPCs (left, green; right, white) surrounding mCherry$^+$ transduced neurons (red) were whole-cell patch clamped and dye-filled with Lucifer yellow (LY; green).

(H–J) The resting membrane potential in OPCs was not different between Gq and control mice. However, increasing neuronal activity increased membrane resistance and decreased the inward K$^+$ conductance in OPCs.

(K) Timeline of the experiment in Kir2.1 mice.

(L–N) The resting membrane potential and inward conductance in OPCs overexpressing Kir2.1 channels were not affected by increased neuronal activity. However, the membrane resistance in OPCs overexpressing Kir2.1 channels slightly increased in Gq mice compared to controls.

(O and P) Timeline of EdU experiments in NG2-EYFP mice and Kir2.1-overexpressing mice with chemogenetically increased neuronal activity.

(Q) To examine OPC proliferation following neuronal activity, slices were stained with DAPI (blue), EdU (white), and EYFP (green). Areas with mCherry$^+$ neurons (red) were imaged. Arrowheads indicate EdU$^+$ proliferating OPCs.

(R) To examine OPC proliferation following neuronal activity in Kir2.1 mice, slices were stained with EdU (green), mCherry to label OPCs overexpressing Kir2.1 channels, and NG2 (white). Arrowheads indicate EdU$^+$ proliferating mCherry$^-$ and mCherry$^+$ OPCs.

(S) Proliferation was increased in OPCs of NG2-EYFP-Gq mice, but not in Kir2.1-Gq mice.

Gray dots represent individual recorded cells (H–J and L–N) or animals (C and S) or brain slices (E). The *p* values are from unpaired two-tailed t tests (C, H–J, and L–N), paired t test (E), or one-way ANOVA (S; top) with Holm-Bonferroni *post hoc* tests (S; bottom). Data are shown as the mean ± SEM.

proliferation (Figures 7O, 7Q, and 7S). Overexpressing Kir2.1 channels in OPCs counteracted the activity-induced change in passive bioelectrical properties (Figures 7K–7N) and prevented the activity-evoked increase in OPC proliferation (Figures 7P, 7R, and 7S). The increased inward K^+ conductance may therefore be a mechanism to ensure that OPCs within the cortex respond only to high neuronal activity that is sufficient to evoke SLICs, which release them from the arrested G1/G0 state by reducing their inward K^+ conductance.

DISCUSSION

We have identified that the inward K^+ conductance in OPCs increases with age, with time of onset differing between brain regions. Similar to other ion channels,[21] OPCs start out with indistinguishable passive bioelectrical properties such as RMP, Rm, and inward K^+ conductance and remain homogeneous for the first postnatal week, after which they begin to diverge. The time at which OPCs acquire functional Kir channels differs greatly between regions, generating a heterogeneity in passive bioelectrical properties. This heterogeneity represents a difference in OPC cell cycle state, with high-K^+-conducting OPCs presumably being arrested in the G1 phase but with the potential for increased neuronal activity to release them back into the cell cycle. These changes in bioelectrical properties can therefore significantly affect how OPCs sense and respond to their environment.

OPCs in areas of high neuronal density can sense submillimolar extracellular K^+ release from active neurons, which coincides with synaptic inputs likely coming from these same neurons. The extracellular K^+ oscillations follow the pattern of neuronal activity and mediate the temporal pattern of SLICs in OPCs. OPCs' sensitivity to these K^+ oscillations is mediated via Kir channels, and the amplitude of SLICs reflects the passive bioelectrical properties of the OPCs more than the $[K^+]_e$, in contrast to previous suggestions.[28,50] It is unlikely that Kir channels in OPCs syphon extracellular K^+ around active neurons, like they do in astrocytes, as OPCs are small cells and are not gap-junction coupled, nor are OPCs with high inward K^+ conductance particularly close to blood vessels, and thus they have no means of releasing K^+ into the bloodstream.

It is more likely that the passive bioelectrical properties serve to regulate how OPCs respond to neuronal activity and their environment. As the increased inward K^+ conductance lowers OPC RMP and resistance, this will work as a filter altering the temporal resolution and summation of synaptic inputs to the OPCs[37,46] and reduce the response to neuronal activity. Indeed, when Kir4.1 is knocked out of hippocampal OPCs[37] or pharmacologically blocked,[46] synaptic inputs are longer and lead to greater depolarization. However, during periods of high neuronal firing rate, the $[K^+]_e$ rises high enough to evoke SLICs in OPCs with high inward K^+ conductance, causing a significantly prolonged depolarization (Figure 3A).[28] Kir channel expression in OPCs in effect alters the threshold and activity pattern for depolarization. Moreover, increased inward K^+ conductance hyperpolarizes the cell membrane and increases the driving force of positive ions likely to lead to increased calcium entry into OPCs during high neuronal activity. Consistent with our findings, OPCs in neuron-rich areas in the zebrafish have transient calcium oscillations[19] with kinetics similar to those found in glioma cells (presumably derived from OPCs) in the mouse cortex, showing SLIC-like currents driven by K^+.[55,56] These glioma SLIC-like events follow synaptic inputs similar to SLICs in OPCs and lead to increased intracellular calcium in response to high neuronal activity (or when neuronal activity is increased pharmacologically in acute brain slices) with spontaneous frequencies similar to those we recorded for SLICs in the same brain region.[55] Hence, it seems that increased Kir channel membrane expression polarizes OPCs to respond preferentially to high synchronous neuronal activity.

The functional change mediated by high inward K^+ conductance early in cortical development has led to speculation that OPCs in neuronal-soma-rich areas have acquired a different cell identity.[28] Like previous studies, we found that inward K^+ conductance in cortical OPCs is prominent by the end of the first month.[28] However, while the onset of increased inward K^+ conductance occurred first in OPCs in areas of high neuronal density, eventually OPCs in all areas tested acquired these channels, with the age of onset varying between regions. This implies an environmental factor regulating channel densities. We find that altering the environment, either by culturing OPCs with a high density of neurons or by increasing neuronal firing rate *in vivo*, can lead to changes in inward K^+ conductance in OPCs. Consistent with this, cortical OPCs, when activated in response to a lesion, reduce their inward conductance, as presumably they have reentered the cell cycle.[33] There are likely to be multiple mechanisms that can induce such passive bioelectrical changes, and these may differ with age and brain region. Overall, the differences in passive bioelectrical properties suggest dynamic cell states rather than a fixed new cell identity.

It is striking that the divergence in passive bioelectrical properties coincides with the start of myelination in the corpus callosum, particularly given that axons of cortical neurons begin to myelinate in the corpus callosum but remain unmyelinated in the cortex for some time. This staggered myelination of the same axons has led to the suggestion that there must be a signal that negatively regulates OPC lineage progression in the cortex.[65] One such signal could be augmentation of Kir channel expression in cortical OPCs that minimizes their response to neuronal activity and keeps them in an extended G1 state or dormant state. This is consistent with the observation that increased inward K^+ conductance in OPCs[66] reduces their response to positive oligodendrocyte lineage fate regulators.[66,67] The existence of two populations of cortical OPCs in young adults, with low and high inward K^+ conductance depending on the environment they are in, is reminiscent of the hypothesized two types of cortical OPCs, where one is a silent OPC kept in an undifferentiated and low proliferative state.[68] Extrapolating from our data, it is conceivable that any signal or channel, for example, G-protein-gated inwardly rectifying K^+ channels, that augments inward K^+ conductance in OPCs could affect their cell cycle regulation and fate.

In newly born animals, both cortical and callosal OPCs have a high proliferation rate and lack Kir channel conductance, like OPCs in which Kir channels have been knocked out.[32] Once OPCs acquire inward K^+ conductance, their proliferation rate is

reduced. This occurs first in high-density neuronal areas such as the cortex and later in other brain regions at different ages. In line with our data, previous studies have shown that OPCs proliferate less in the cortex than in the corpus callosum of young adult animals[20] and in areas rich in neuronal cell bodies compared to axonal-rich areas in the zebrafish gray matter.[19] Our description of the expression of inward K^+ channels in OPCs correlates with the reported proliferation difference between white and gray matter and aged OPCs,[17,18] and a similar correlation has been found in hippocampal OPCs, where an increase in inward K^+ conductance correlated with a decrease in proliferation with age.[27] Indeed, we find that OPCs in G1/G0 have higher membrane K^+ conductance and lower Rm, like astrocyte progenitors in the G1/G0 phase of the cell cycle,[69] and when we genetically increase Kir channel expression in OPCs, proliferation is reduced. This is consistent with inward K^+ conductance regulating the length of the G1 phase or cell cycle exit in astrocytes[70,71] and other proliferative progenitor populations.[39,72] In line with this, knocking out Kir4.1 channels in OPCs leads to precocious cell cycle exit and earlier differentiation into myelinating oligodendrocytes.[36] Although no clear difference in proliferation is reported when Kir4.1 is deleted from OPCs,[32] a subtle regional difference in proliferation rate seems to be lost.[37] The differences in the observed effect of knocking out Kir4.1 in OPCs may be dependent on age and brain region studied, given the differences in expression with these channels. A recent study on the role of calcium-impermeable AMPA receptors highlights the importance of understanding endogenous expression profile changes with age when selecting time points for intervention.[73]

In summary, our data show that OPCs start with homogeneous passive bioelectrical properties but become functionally heterogeneous both between and within brain regions. However, by middle age, their passive bioelectrical properties become more homogeneous again. Our findings suggest that the functional heterogeneity is plastic, and although it may impact OPC interaction with neuronal network function, it seems not to indicate that OPCs have become different cell types or alternatives to oligodendrocyte precursors nor that the changes in channel membrane density are irreversible. Our data support environmental regulation turning on, or accelerating, Kir channel expression in OPCs, and environmental changes such as those arising from increased neuronal activity are sufficient to alter OPCs' passive bioelectrical properties and push them back into the cell cycle. This heterogeneity in bioelectrical properties underlies differences in proliferation potential and indicates a state of OPCs that are in an extended G1/G0 phase of the cell cycle, from which they can be released. Understanding further how OPCs' bioelectrical properties are regulated and how these in turn modulate cell cycle and differentiation may enable us to find ways to manipulate OPC cell states to promote myelin maintenance and regeneration with age.

Limitations of the study

We used a combination of endogenous expression and overexpression of Kir channels to understand the role of inward K^+ conductance in OPCs. We find that inward K^+ conductance strongly correlates with cell cycle arrest and proliferation rate. OPCs vary in their expression of Kir channels at different ages and within brain regions. Although cells with high inward K^+ conductance proliferate less than cells with low conductance, the relationship between the magnitude of inward K^+ conductance and cell cycle length remains undefined. Experimental tools that enable dynamic genetic regulation of both Kir2.1 and Kir4.1 expression in OPCs would allow us to directly address this question and distinguish between environmental signals and Kir channel function at any given age or region.

One outstanding question is how neuronal activity regulates Kir channel expression in OPCs. We found that increasing activity seemed to release OPCs from cell cycle arrest by reducing inward K^+ conductance, but the mechanism is still unclear. More sophisticated experimental tools are required to track Kir channel function in single cells through cell cycle progression and to determine how Kir channel expression is regulated either by neuronal activity or during the cell cycle in OPCs. Our finding that basic bioelectrical properties may underlie functional OPC heterogeneity raises an interesting avenue for further studies to determine how these properties affect OPC differentiation, myelination, and perhaps remyelination.

RESOURCE AVAILABILITY

Lead contact

Requests for further information and resources and reagents should be directed to, and will be fulfilled, by the lead contact, Ragnhildur T. Káradóttir (rk385@cam.ac.uk).

Materials availability

This study did not generate new reagents.

Data and code availability

- All data reported in this paper will be shared by the lead contact upon request.
- This paper does not report original code.
- Any additional information required to reanalyze the data reported in this paper is available from the lead contact upon request.

ACKNOWLEDGMENTS

We thank Jacqueline Trotter (Johannes Gutenberg-University, Mainz, Germany) for the NG2-EYFP mice, William D. Richardson (University College London, London, UK) for the PDGFRα-CreER^{T2} mice, and Guillermina Lopez-Bendito (Instituto de Neurosciencias, Alicante, Spain) for the *R26*$^{Kir2.1\text{-}mCherry}$ mice. We thank Stephanie Hall and all the members of the Káradóttir laboratory for comments on the manuscript. We acknowledge the support of the Cambridge Stem Cell Institute core facility staff, in particular the imaging facility, and University Biomedical Services. This project received funding from the European Research Council under the European Union's Horizon 2020 Research And Innovation Programme (grant agreement no. 771411; R.T.K., A.V., H.P., and Y.K.); a Wellcome Pathfinder Award (204488/Z/16/Z, R.T.K. and H.P.), studentship (102160/Z/13/Z; Y.K.), and centre award (203151/Z/16/Z, 203151/A/16/Z); a UKRI Medical Research Council, studentship (S.O.S.), programme grant (MR/Y014537/1; R.T.K., O.d.F.), and centre award (MC_PC_17230); the Gates Cambridge Trust, Gates Scholarship (S.S.); an MS Society Centre of Excellence grant, Cambridge Centre for Myelin Repair (132; R.T.K. and O.d.F.); a Fonds de Recherche du Québec – Santé scholarship (Y.K.); a Cambridge Commonwealth European & International Trust scholarship (Y.K.); the Lister Institute, Research Prize (R.T.K. and S.O.S.); the Newton Trust project and emergency grant (19.23(t) and 21.07 (g); A.V. and

R.T.K.); the Career Support fund of Cambridge University (A.V.); and Charles University (PRIMUS/21/MED/005; H.P.). For the purpose of open access, the authors have applied a CC BY public copyright license to any Author Accepted Manuscript version arising from this submission.

AUTHOR CONTRIBUTIONS

Conceptualization, R.T.K., S.S., and H.P.; investigation, H.P., S.S., Y.K., M.M., A.V., O.d.F., Y.T.N., B.V.V., S.O.S., and C.T.; data analysis: H.P., S.S., Y.K., S.O.S., M.M. A.V., and R.T.K.; writing, R.T.K., H.P., and Y.K., and all authors commented on the manuscript. Funding acquisition, resources, and supervision, R.T.K.

DECLARATION OF INTERESTS

The authors have no competing interests to declare.

STAR★METHODS

Detailed methods are provided in the online version of this paper and include the following:

- KEY RESOURCES TABLE
- EXPERIMENTAL MODEL AND STUDY PARTICIPANT DETAILS
- METHOD DETAILS
  - Brain slices preparation
  - Solutions
  - Electrophysiology
  - Fabrication of K^+-selective microelectrodes and measurement of extracellular K^+ concentration
  - Immunohistochemistry
  - Increasing neuronal activity by DREADDs
  - Neuron-OPC co-cultures
  - Kir4.1 channel overexpression in OPC cultures
- QUANTIFICATION AND STATISTICAL ANALYSIS

SUPPLEMENTAL INFORMATION

Supplemental information can be found online at https://doi.org/10.1016/j.celrep.2024.114873.

Received: January 10, 2023
Revised: July 12, 2024
Accepted: September 27, 2024
Published: October 17, 2024

REFERENCES

1. Bonetto, G., Belin, D., and Káradóttir, R.T. (2021). Myelin: A gatekeeper of activity-dependent circuit plasticity? Science *374*, eaba6905. https://doi.org/10.1126/science.aba6905.
2. Nave, K.A. (2010). Myelination and the trophic support of long axons. Nat. Rev. Neurosci. *11*, 275–283. https://doi.org/10.1038/nrn2797.
3. de Faria, O., Jr., Pivonkova, H., Varga, B., Timmler, S., Evans, K.A., and Káradóttir, R.T. (2021). Periods of synchronized myelin changes shape brain function and plasticity. Nat. Neurosci. *24*, 1508–1521. https://doi.org/10.1038/s41593-021-00917-2.
4. Gautier, H.O.B., Evans, K.A., Volbracht, K., James, R., Sitnikov, S., Lundgaard, I., James, F., Lao-Peregrin, C., Reynolds, R., Franklin, R.J.M., and Káradóttir, R.T. (2015). Neuronal activity regulates remyelination via glutamate signalling to oligodendrocyte progenitors. Nat. Commun. *6*, 8518. https://doi.org/10.1038/ncomms9518.
5. Barres, B.A., and Raff, M.C. (1993). Proliferation of oligodendrocyte precursor cells depends on electrical activity in axons. Nature *361*, 258–260. https://doi.org/10.1038/361258a0.
6. Gibson, E.M., Purger, D., Mount, C.W., Goldstein, A.K., Lin, G.L., Wood, L.S., Inema, I., Miller, S.E., Bieri, G., Zuchero, J.B., et al. (2014). Neuronal Activity Promotes Oligodendrogenesis and Adaptive Myelination in the Mammalian Brain. Science *344*, 1252304. https://doi.org/10.1126/science.1252304.
7. Mitew, S., Gobius, I., Fenlon, L.R., McDougall, S.J., Hawkes, D., Xing, Y.L., Bujalka, H., Gundlach, A.L., Richards, L.J., Kilpatrick, T.J., et al. (2018). Pharmacogenetic stimulation of neuronal activity increases myelination in an axon-specific manner. Nat. Commun. *9*, 306. https://doi.org/10.1038/s41467-017-02719-2.
8. Ruckh, J.M., Zhao, J.-W., Shadrach, J.L., van Wijngaarden, P., Rao, T.N., Wagers, A.J., and Franklin, R.J.M. (2012). Rejuvenation of Regeneration in the Aging Central Nervous System. Cell Stem Cell *10*, 96–103. https://doi.org/10.1016/j.stem.2011.11.019.
9. Hill, R.A., Li, A.M., and Grutzendler, J. (2018). Lifelong cortical myelin plasticity and age-related degeneration in the live mammalian brain. Nat. Neurosci. *21*, 683–695. https://doi.org/10.1038/s41593-018-0120-6.
10. Chen, J.-F., Liu, K., Hu, B., Li, R.-R., Xin, W., Chen, H., Wang, F., Chen, L., Li, R.-X., Ren, S.-Y., et al. (2021). Enhancing myelin renewal reverses cognitive dysfunction in a murine model of Alzheimer's disease. Neuron *109*, 2292–2307.e5. https://doi.org/10.1016/j.neuron.2021.05.012.
11. Iram, T., Kern, F., Kaur, A., Myneni, S., Morningstar, A.R., Shin, H., Garcia, M.A., Yerra, L., Palovics, R., Yang, A.C., et al. (2022). Young CSF restores oligodendrogenesis and memory in aged mice via Fgf17. Nature *605*, 509–515. https://doi.org/10.1038/s41586-022-04722-0.
12. Habes, M., Erus, G., Toledo, J.B., Zhang, T., Bryan, N., Launer, L.J., Rosseel, Y., Janowitz, D., Doshi, J., Van der Auwera, S., et al. (2016). White matter hyperintensities and imaging patterns of brain ageing in the general population. Brain *139*, 1164–1179. https://doi.org/10.1093/brain/aww008.
13. Dawson, M.R.L., Polito, A., Levine, J.M., and Reynolds, R. (2003). NG2-expressing glial progenitor cells: an abundant and widespread population of cycling cells in the adult rat CNS. Mol. Cell. Neurosci. *24*, 476–488. https://doi.org/10.1016/S1044-7431(03)00210-0.
14. Mason, J.L., and Goldman, J.E. (2002). A2B5+ and O4+ Cycling Progenitors in the Adult Forebrain White Matter Respond Differentially to PDGF-AA, FGF-2, and IGF-1. Mol. Cell. Neurosci. *20*, 30–42. https://doi.org/10.1006/mcne.2002.1114.
15. Lentferink, D.H., Jongsma, J.M., Werkman, I., and Baron, W. (2018). Grey matter OPCs are less mature and less sensitive to IFNγ than white matter OPCs: Consequences for remyelination. Sci. Rep. *8*, 2113. https://doi.org/10.1038/s41598-018-19934-6.
16. Marques, S., van Bruggen, D., Vanichkina, D.P., Floriddia, E.M., Munguba, H., Väremo, L., Giacomello, S., Falcão, A.M., Meijer, M., Björklund, Å.K., et al. (2018). Transcriptional Convergence of Oligodendrocyte Lineage Progenitors during Development. Dev. Cell *46*, 504–517.e7. https://doi.org/10.1016/j.devcel.2018.07.005.
17. Psachoulia, K., Jamen, F., Young, K.M., and Richardson, W.D. (2009). Cell cycle dynamics of NG2 cells in the postnatal and ageing brain. Neuron Glia Biol. *5*, 57–67. https://doi.org/10.1017/S1740925X09990354.
18. Young, K.M., Psachoulia, K., Tripathi, R.B., Dunn, S.-J., Cossell, L., Attwell, D., Tohyama, K., and Richardson, W.D. (2013). Oligodendrocyte Dynamics in the Healthy Adult CNS: Evidence for Myelin Remodeling. Neuron *77*, 873–885. https://doi.org/10.1016/j.neuron.2013.01.006.
19. Marisca, R., Hoche, T., Agirre, E., Hoodless, L.J., Barkey, W., Auer, F., Castelo-Branco, G., and Czopka, T. (2020). Functionally distinct subgroups of oligodendrocyte precursor cells integrate neural activity and execute myelin formation. Nat. Neurosci. *23*, 363–374. https://doi.org/10.1038/s41593-019-0581-2.
20. Viganò, F., Möbius, W., Götz, M., and Dimou, L. (2013). Transplantation reveals regional differences in oligodendrocyte differentiation in the adult brain. Nat. Neurosci. *16*, 1370–1372. https://doi.org/10.1038/nn.3503.
21. Spitzer, S.O., Sitnikov, S., Kamen, Y., Evans, K.A., Kronenberg-Versteeg, D., Dietmann, S., de Faria, O., Agathou, S., and Káradóttir, R.T. (2019).

Oligodendrocyte Progenitor Cells Become Regionally Diverse and Heterogeneous with Age. Neuron *101*, 459–471.e5. https://doi.org/10.1016/j.neuron.2018.12.020.

22. Chittajallu, R., Aguirre, A., and Gallo, V. (2004). NG2-positive cells in the mouse white and grey matter display distinct physiological properties. J. Physiol. *561*, 109–122. https://doi.org/10.1113/jphysiol.2004.074252.
23. Clarke, L.E., Young, K.M., Hamilton, N.B., Li, H., Richardson, W.D., and Attwell, D. (2012). Properties and Fate of Oligodendrocyte Progenitor Cells in the Corpus Callosum, Motor Cortex, and Piriform Cortex of the Mouse. J. Neurosci. *32*, 8173–8185. https://doi.org/10.1523/JNEUROSCI.0928-12.2012.
24. De Biase, L.M., Nishiyama, A., and Bergles, D.E. (2010). Excitability and Synaptic Communication within the Oligodendrocyte Lineage. J. Neurosci. *30*, 3600–3611. https://doi.org/10.1523/JNEUROSCI.6000-09.2010.
25. Kamen, Y., Pivonkova, H., Evans, K.A., and Káradóttir, R.T. (2022). A Matter of State: Diversity in Oligodendrocyte Lineage Cells. Neuroscientist *28*, 144–162. https://doi.org/10.1177/1073858420987208.
26. Lim, L., Mi, D., Llorca, A., and Marín, O. (2018). Development and Functional Diversification of Cortical Interneurons. Neuron *100*, 294–313. https://doi.org/10.1016/j.neuron.2018.10.009.
27. Moshrefi-Ravasdjani, B., Dublin, P., Seifert, G., Jennissen, K., Steinhäuser, C., Kafitz, K.W., and Rose, C.R. (2017). Changes in the proliferative capacity of NG2 cell subpopulations during postnatal development of the mouse hippocampus. Brain Struct. Funct. *222*, 831–847. https://doi.org/10.1007/s00429-016-1249-2.
28. Maldonado, P.P., Vélez-Fort, M., Levavasseur, F., and Angulo, M.C. (2013). Oligodendrocyte Precursor Cells Are Accurate Sensors of Local K+ in Mature Gray Matter. J. Neurosci. *33*, 2432–2442. https://doi.org/10.1523/JNEUROSCI.1961-12.2013.
29. Bedner, P., Jabs, R., and Steinhäuser, C. (2020). Properties of human astrocytes and NG2 glia. Glia *68*, 756–767. https://doi.org/10.1002/glia.23725.
30. Duncan, G.J., Simkins, T.J., and Emery, B. (2021). Neuron-Oligodendrocyte Interactions in the Structure and Integrity of Axons. Front. Cell Dev. Biol. *9*, 653101. https://doi.org/10.3389/fcell.2021.653101.
31. Pérez-Samartín, A., Garay, E., Moctezuma, J.P.H., Cisneros-Mejorado, A., Sánchez-Gómez, M.V., Martel-Gallegos, G., Robles-Martínez, L., Canedo-Antelo, M., Matute, C., and Arellano, R.O. (2017). Inwardly Rectifying K+ Currents in Cultured Oligodendrocytes from Rat Optic Nerve are Insensitive to pH. Neurochem. Res. *42*, 2443–2455. https://doi.org/10.1007/s11064-017-2242-8.
32. Larson, V.A., Mironova, Y., Vanderpool, K.G., Waisman, A., Rash, J.E., Agarwal, A., and Bergles, D.E. (2018). Oligodendrocytes control potassium accumulation in white matter and seizure susceptibility. Elife *7*, 1–33. https://doi.org/10.7554/eLife.34829.
33. Song, F., Hong, X., Cao, J., Ma, G., Han, Y., Cepeda, C., Kang, Z., Xu, T., Duan, S., Wan, J., and Tong, X. (2018). Kir4.1 channels in NG2-glia play a role in development, potassium signaling, and ischemia-related myelin loss. Commun. Biol. *1*, 80. https://doi.org/10.1038/s42003-018-0083-x.
34. Poopalasundaram, S., Knott, C., Shamotienko, O.G., Foran, P.G., Dolly, J.O., Ghiani, C.A., Gallo, V., and Wilkin, G.P. (2000). Glial heterogeneity in expression of the inwardly rectifying K+ channel, Kir4.1, in adult rat CNS. Glia *30*, 362–372. https://doi.org/10.1002/(SICI)1098-1136(200006)30:4<362::AID-GLIA50>3.0.CO;2-4.
35. Brasko, C., and Butt, A. (2018). Expression of Kir2.1 Inward Rectifying Potassium Channels in Optic Nerve Glia: Evidence for Heteromeric Association with Kir4.1 and Kir5.1. Neuroglia *1*, 176–187. https://doi.org/10.3390/neuroglia1010012.
36. Schirmer, L., Möbius, W., Zhao, C., Cruz-Herranz, A., Ben Haim, L., Cordano, C., Shiow, L.R., Kelley, K.W., Sadowski, B., Timmons, G., et al. (2018). Oligodendrocyte-encoded Kir4.1 function is required for axonal integrity. Elife *7*, 1–21. https://doi.org/10.7554/eLife.36428.
37. Timmermann, A., Tascio, D., Jabs, R., Boehlen, A., Domingos, C., Skubal, M., Huang, W., Kirchhoff, F., Henneberger, C., Bilkei-Gorzo, A., et al. (2023). Dysfunction of NG2 glial cells affects neuronal plasticity and behavior. Glia *71*, 1481–1501. https://doi.org/10.1002/glia.24352.
38. Sakry, D., Neitz, A., Singh, J., Frischknecht, R., Marongiu, D., Biname, F., Perera, S.S., Endres, K., Lutz, B., Radyushkin, K., et al. (2014). Oligodendrocyte Precursor Cells Modulate the Neuronal Network by Activity-Dependent Ectodomain Cleavage of Glial NG2. PLoS Biol. *12*, e1001993. https://doi.org/10.1371/journal.pbio.1001993.
39. Urrego, D., Tomczak, A.P., Zahed, F., Stühmer, W., and Pardo, L.A. (2014). Potassium channels in cell cycle and cell proliferation. Phil Trans R Soc B *369*, 20130094. https://doi.org/10.1098/rstb.2013.0094.
40. Gallo, V., Zhou, J.M., McBain, C.J., Wright, P., Knutson, P.L., and Armstrong, R.C. (1996). Oligodendrocyte progenitor cell proliferation and lineage progression are regulated by glutamate receptor-mediated K+ channel block. J. Neurosci. *16*, 2659–2670. https://doi.org/10.1523/JNEUROSCI.16-08-02659.1996.
41. Ghiani, C.A., Yuan, X., Eisen, A.M., Knutson, P.L., DePinho, R.A., McBain, C.J., and Gallo, V. (1999). Voltage-activated K+ channels and membrane depolarization regulate accumulation of the cyclin-dependent kinase inhibitors p27(Kip1) and p21(CIP1) in glial progenitor cells. J. Neurosci. *19*, 5380–5392. https://doi.org/10.1523/JNEUROSCI.19-13-05380.1999.
42. Karram, K., Goebbels, S., Schwab, M., Jennissen, K., Seifert, G., Steinhäuser, C., Nave, K.-A., and Trotter, J. (2008). NG2-expressing cells in the nervous system revealed by the NG2-EYFP-knockin mouse. Genesis *46*, 743–757. https://doi.org/10.1002/dvg.20440.
43. Sturrock, R.R. (1980). Myelination of the Mouse Corpus Callosum. Neuropathol. Appl. Neurobiol. *6*, 415–420. https://doi.org/10.1111/j.1365-2990.1980.tb00219.x.
44. Tripathi, R.B., Jackiewicz, M., McKenzie, I.A., Kougioumtzidou, E., Grist, M., Richardson, W.D., Tripathi, R.B., Jackiewicz, M., Grist, M., McKenzie, I.A., et al. (2017). Remarkable Stability of Myelinating Oligodendrocytes in Mice. Cell Rep. *21*, 316–323. https://doi.org/10.1016/j.celrep.2017.09.050.
45. Hughes, E.G., Kang, S.H., Fukaya, M., and Bergles, D.E. (2013). Oligodendrocyte progenitors balance growth with self-repulsion to achieve homeostasis in the adult brain. Nat. Neurosci. *16*, 668–676. https://doi.org/10.1038/nn.3390.
46. Chan, C.-F., Kuo, T.-W., Weng, J.-Y., Lin, Y.-C., Chen, T.-Y., Cheng, J.-K., and Lien, C.-C. (2013). Ba2+- and bupivacaine-sensitive background K+ conductances mediate rapid EPSP attenuation in oligodendrocyte precursor cells. J. Physiol. *591*, 4843–4858. https://doi.org/10.1113/jphysiol.2013.257113.
47. Aram, J.A., and Lodge, D. (1988). Validation of a neocortical slice preparation for the study of epileptiform activity. J. Neurosci. Methods *23*, 211–224. https://doi.org/10.1016/0165-0270(88)90005-2.
48. Sykova, E. (1981). K+ changes in the extracellular space of the spinal cord and their physiological role. J. Exp. Biol. *95*, 93–109. https://doi.org/10.1242/jeb.95.1.93.
49. Silva, L.R., Amitai, Y., and Connors, B.W. (1991). Intrinsic Oscillations of Neocortex Generated by Layer 5 Pyramidal Neurons. Science *251*, 432–435. https://doi.org/10.1126/science.1824881.
50. Kettenmann, H., Sonnhof, U., and Schachner, M. (1983). Exclusive potassium dependence of the membrane potential in cultured mouse oligodendrocytes. J. Neurosci. *3*, 500–505. https://doi.org/10.1523/JNEUROSCI.03-03-00500.1983.
51. Karnup, S., and Stelzer, A. (2001). Seizure-like activity in the disinhibited CA1 minislice of adult guinea-pigs. J. Physiol. *532*, 713–730. https://doi.org/10.1111/j.1469-7793.2001.0713e.x.
52. Bhave, G., Lonergan, D., Chauder, B.A., and Denton, J.S. (2010). Small-molecule modulators of inward rectifier K^{+} channels: recent advances and future possibilities. Future Med. Chem. *2*, 757–774. https://doi.org/10.4155/fmc.10.179.

53. Kharade, S.V., Kurata, H., Bender, A.M., Blobaum, A.L., Figueroa, E.E., Duran, A., Kramer, M., Days, E., Vinson, P., Flores, D., et al. (2018). Discovery, characterization, and effects on renal fluid and electrolyte excretion of the Kir4.1 potassium channel pore blocker, VU0134992. Mol. Pharmacol. *94*, 926–937. https://doi.org/10.1124/mol.118.112359.

54. Wang, H.R., Wu, M., Yu, H., Long, S., Stevens, A., Engers, D.W., Sackin, H., Daniels, J.S., Dawson, E.S., Hopkins, C.R., et al. (2011). Selective inhibition of the K ir2 family of inward rectifier potassium channels by a small molecule probe: The discovery, SAR, and pharmacological characterization of ML133. ACS Chem. Biol. *6*, 845–856. https://doi.org/10.1021/cb200146a.

55. Venkataramani, V., Tanev, D.I., Strahle, C., Studier-Fischer, A., Fankhauser, L., Kessler, T., Körber, C., Kardorff, M., Ratliff, M., Xie, R., et al. (2019). Glutamatergic synaptic input to glioma cells drives brain tumour progression. Nature *573*, 532–538. https://doi.org/10.1038/s41586-019-1564-x.

56. Venkatesh, H.S., Morishita, W., Geraghty, A.C., Silverbush, D., Gillespie, S.M., Arzt, M., Tam, L.T., Espenel, C., Ponnuswami, A., Ni, L., et al. (2019). Electrical and synaptic integration of glioma into neural circuits. Nature *573*, 539–545. https://doi.org/10.1038/s41586-019-1563-y.

57. Pivonkova, H., Benesova, J., Butenko, O., Chvatal, A., and Anderova, M. (2010). Impact of global cerebral ischemia on K+ channel expression and membrane properties of glial cells in the rat hippocampus. Neurochem. Int. *57*, 783–794. https://doi.org/10.1016/j.neuint.2010.08.016.

58. Fakler, B., Bond, C.T., Adelman, J.P., and Ruppersberg, J.P. (1996). Heterooligomeric assembly of inward-rectifier K+ channels from subunits of different subfamilies: K(ir)2.1 (IRK1) and K(ir)4.1 (BIR10). Pflügers Archiv *433*, 77–83. https://doi.org/10.1007/s004240050251.

59. Hibino, H., Inanobe, A., Furutani, K., Murakami, S., Findlay, I., and Kurachi, Y. (2010). Inwardly Rectifying Potassium Channels: Their Structure, Function, and Physiological Roles. Physiol. Rev. *90*, 291–366. https://doi.org/10.1152/physrev.00021.2009.

60. Moreno-Juan, V., Filipchuk, A., Antón-Bolaños, N., Mezzera, C., Gezelius, H., Andrés, B., Rodríguez-Malmierca, L., Susín, R., Schaad, O., Iwasato, T., et al. (2017). Prenatal thalamic waves regulate cortical area size prior to sensory processing. Nat. Commun. *8*, 14172. https://doi.org/10.1038/ncomms14172.

61. Madisen, L., Zwingman, T.A., Sunkin, S.M., Oh, S.W., Zariwala, H.A., Gu, H., Ng, L.L., Palmiter, R.D., Hawrylycz, M.J., Jones, A.R., et al. (2010). A robust and high-throughput Cre reporting and characterization system for the whole mouse brain. Nat. Neurosci. *13*, 133–140. https://doi.org/10.1038/nn.2467.

62. Alexander, G.M., Rogan, S.C., Abbas, A.I., Armbruster, B.N., Pei, Y., Allen, J.A., Nonneman, R.J., Hartmann, J., Moy, S.S., Nicolelis, M.A., et al. (2009). Remote Control of Neuronal Activity in Transgenic Mice Expressing Evolved G Protein-Coupled Receptors. Neuron *63*, 27–39. https://doi.org/10.1016/j.neuron.2009.06.014.

63. Gomez, J.L., Bonaventura, J., Lesniak, W., Mathews, W.B., Sysa-Shah, P., Rodriguez, L.A., Ellis, R.J., Richie, C.T., Harvey, B.K., Dannals, R.F., et al. (2017). Chemogenetics revealed: DREADD occupancy and activation via converted clozapine. Science *357*, 503–507. https://doi.org/10.1126/science.aan2475.

64. Carcea, I., Caraballo, N.L., Marlin, B.J., Ooyama, R., Riceberg, J.S., Mendoza Navarro, J.M., Opendak, M., Diaz, V.E., Schuster, L., Alvarado Torres, M.I., et al. (2021). Oxytocin neurons enable social transmission of maternal behaviour. Nature *596*, 553–557. https://doi.org/10.1038/s41586-021-03814-7.

65. Mallon, B.S., Shick, H.E., Kidd, G.J., and Macklin, W.B. (2002). Proteolipid Promoter Activity Distinguishes Two Populations of NG2-Positive Cells throughout Neonatal Cortical Development. J. Neurosci. *22*, 876–885. https://doi.org/10.1523/JNEUROSCI.22-03-00876.2002.

66. Knutson, P., Ghiani, C.A., Zhou, J.M., Gallo, V., and McBain, C.J. (1997). K+ channel expression and cell proliferation are regulated by intracellular sodium and membrane depolarization in oligodendrocyte progenitor cells. J. Neurosci. *17*, 2669–2682. https://doi.org/10.1523/JNEUROSCI.17-08-02669.1997.

67. Hill, R.A., Patel, K.D., Medved, J., Reiss, A.M., and Nishiyama, A. (2013). NG2 cells in white matter but not gray matter proliferate in response to PDGF. J. Neurosci. *33*, 19393–19395. https://doi.org/10.1523/JNEUROSCI.2001-12.2013.

68. Hermann, A., Brandt, M.D., Loewenbrück, K.F., and Storch, A. (2010). "Silenced" polydendrocytes: a new cell type within the oligodendrocyte progenitor cell population? Cell Tissue Res. *340*, 45–50. https://doi.org/10.1007/s00441-010-0940-5.

69. MacFarlane, S.N., and Sontheimer, H. (2000). Changes in ion channel expression accompany cell cycle progression of spinal cord astrocytes. Glia *30*, 39–48. https://doi.org/10.1002/(SICI)1098-1136(200003)30:1<39::AID-GLIA5>3.0.CO;2-S.

70. Higashimori, H., and Sontheimer, H. (2007). Role of Kir4.1 channels in growth control of glia. Glia *55*, 1668–1679. https://doi.org/10.1002/glia.20574.

71. Olsen, M.L., and Sontheimer, H. (2008). Functional implications for Kir4.1 channels in glial biology: from K^+ buffering to cell differentiation. J. Neurochem. *107*, 589–601. https://doi.org/10.1111/j.1471-4159.2008.05615.x.

72. Blackiston, D.J., McLaughlin, K.A., and Levin, M. (2009). Bioelectric controls of cell proliferation: Ion channels, membrane voltage and the cell cycle. Cell Cycle *8*, 3527–3536. https://doi.org/10.4161/cc.8.21.9888.

73. Khawaja, R.R., Agarwal, A., Fukaya, M., Jeong, H.-K., Gross, S., Gonzalez-Fernandez, E., Soboloff, J., Bergles, D.E., and Kang, S.H. (2021). GluA2 overexpression in oligodendrocyte progenitors promotes postinjury oligodendrocyte regeneration. Cell Rep. *35*, 109147. https://doi.org/10.1016/j.celrep.2021.109147.

74. Galvan, A., Raper, J., Hu, X., Pare, J.F., Bonaventura, J., Richie, C.T., Michaelides, M., Mueller, S.A.L., Roseboom, P.H., Oler, J.A., et al. (2019). Ultrastructural localization of DREADDs in monkeys. Eur. J. Neurosci. *50*, 2801–2813. https://doi.org/10.1111/ejn.14429.

75. Rivers, L.E., Young, K.M., Rizzi, M., Jamen, F., Psachoulia, K., Wade, A., Kessaris, N., and Richardson, W.D. (2008). PDGFRA/NG2 glia generate myelinating oligodendrocytes and piriform projection neurons in adult mice. Nat. Neurosci. *11*, 1392–1401. https://doi.org/10.1038/nn.2220.

76. Mort, R.L., Ford, M.J., Sakaue-Sawano, A., Lindstrom, N.O., Casadio, A., Douglas, A.T., Keighren, M.A., Hohenstein, P., Miyawaki, A., and Jackson, I.J. (2014). *Fucci2a:* A bicistronic cell cycle reporter that allows Cre mediated tissue specific expression in mice. Cell Cycle *13*, 2681–2696. https://doi.org/10.4161/15384101.2015.945381.

77. Chen, Y., Spitzer, S., Agathou, S., Karadottir, R.T., and Smith, A. (2017). Gene Editing in Rat Embryonic Stem Cells to Produce In Vitro Models and In Vivo Reporters. Stem Cell Rep. *9*, 1262–1274. https://doi.org/10.1016/j.stemcr.2017.09.005.

78. Agathou, S., and Káradóttir, R.T. (2019). Whole-Cell Patch Clamp Recordings from Oligodendrocyte Lineage Cells in Brain Slices. Methods Mol. Biol. *1936*, 141–168. https://doi.org/10.1007/978-1-4939-9072-6_9.

79. Kamen, Y., and Káradóttir, R.T. (2021). Combining whole-cell patch clamp and dye loading in acute brain slices with bulk RNA sequencing in embryonic to aged mice. STAR Protoc. *2*, 100439. https://doi.org/10.1016/j.xpro.2021.100439.

80. Káradóttir, R., and Attwell, D. (2006). Combining patch-clamping of cells in brain slices with immunocytochemical labeling to define cell type and developmental stage. Nat. Protoc. *1*, 1977–1986. https://doi.org/10.1038/nprot.2006.261.

81. Lundgaard, I., Luzhynskaya, A., Stockley, J.H., Wang, Z., Evans, K.A., Swire, M., Volbracht, K., Gautier, H.O.B., Franklin, R.J.M., et al.; Charles

CellPress
OPEN ACCESS

Ffrench-Constant (2013). Neuregulin and BDNF Induce a Switch to NMDA Receptor-Dependent Myelination by Oligodendrocytes. PLoS Biol. *11*, e1001743. https://doi.org/10.1371/journal.pbio.1001743.

82. Milner, R., Edwards, G., Streuli, C., and Ffrench, C.-C. (1996). A Role in Migration for the αVβ1 Integrin Expressed on Oligodendrocyte Precursors. Clin. Sci. *91*, 18P. https://doi.org/10.1042/cs091018p.

83. McCarthy, K.D., and de Vellis, J. (1980). Preparation of separate astroglial and oligodendroglial cell cultures from rat cerebral tissue. J. Cell Biol. *85*, 890–902. https://doi.org/10.1083/jcb.85.3.890.

84. Stockley, J.H., Evans, K., Matthey, M., Volbracht, K., Agathou, S., Mukanowa, J., Burrone, J., and Káradóttir, R.T. (2017). Surpassing light-induced cell damage in vitro with novel cell culture media. Sci. Rep. *7*, 849. https://doi.org/10.1038/s41598-017-00829-x.

STAR★METHODS

KEY RESOURCES TABLE

REAGENT or RESOURCE	SOURCE	IDENTIFIER
**Antibodies**		
Chicken anti-GFP	Abcam	Cat#ab13970; RRID:AB_300798
Rabbit anti-NG2 Chondroitin Sulfate Proteoglycan	Millipore	Cat#AB5320; RRID:AB_11213678
Rabbit anti-NG2 [EPR23976-145]	Abcam	Cat#ab275024; RRID:AB_2922401
Rabbit anti-NeuN	Millipore	Cat#ABN78; RRID:AB_10807945
Mouse anti-NeuN	Chemicon	Cat#MAB377; RRID:AB_2298772
Rabbit anti-mCherry	Abcam	Cat#ab167453; RRID: AB_2571870
Chicken anti-mCherry	Abcam	Cat#ab205402; RRID:AB_2722769
Rabbit anti-Ki67 [SP6]	Abcam	Cat#ab16667; RRID: AB_302459
Goat Anti-Chicken IgY H&L (Alexa Fluor 488)	Abcam	Cat#ab150169; RRID:AB_2636803
Goat Anti-Chicken IgY H&L (Alexa Fluor 568)	Abcam	Cat#ab175477; RRID: AB_3076392
Invitrogen Goat anti-Chicken IgY (H+L) Secondary Antibody, Alexa Fluor 647	ThermoFisher Scientific	Cat#A-21449; RRID: AB_2535866
Goat anti-Mouse IgG (H+L) Highly Cross-Adsorbed Secondary Antibody, Alexa Fluor 555	ThermoFisher Scientific	Cat# A-21424; RRID:AB_141780
Goat anti Mouse IgG (H+L) Highly Cross Adsorbed Secondary Antibody, Alexa Fluor 647	ThermoFisher Scientific	Cat#A21236; RRID:AB_2535805
Goat anti-Rabbit IgG (H+L) Cross-Adsorbed Secondary Antibody, Alexa Fluor 488	ThermoFisher Scientific	Cat#A11008; RRID:AB_143165
Goat anti-Rabbit IgG (H+L) Highly Cross-Adsorbed Secondary Antibody, Alexa Fluor 568	ThermoFisher Scientific	Cat#A-11036; RRID:AB_10563566
Invitrogen Goat anti-Rabbit IgG (H+L) Highly Cross-Adsorbed Secondary Antibody, Alexa Fluor 647	ThermoFisher Scientific	Cat# A-21245; RRID:AB_2535813
**Bacterial and virus strains**		
p-AAV8-hSyn-hM3D(Gq)-mCherry	Bryan Roth	Addgene 50474-AAV8
pAAV8-hSyn-mCherry	Karl Deisseroth	Addgene 114472-AAV8
pAAV5-SYN1-HA-hM3D(Gq)	Galvan et al.[74]	Addgene 121539-AAV5
pAAV5-hSyn-EGFP	Bryan Roth	Addgene 50465-AAV5
**Chemicals, peptides, and recombinant proteins**		
NaCl	Sigma-Aldrich	Cat#S7653
KCl	Sigma-Aldrich	Cat#P3911
$NaHCO_3$	Sigma-Aldrich	Cat#S5761
NaH_2PO_4	Fisher Scientific	Cat#S/3760/53
$CaCl_2$	VWR	Cat#21114
$MgCl_2$	Fisher Scientific	Cat#15656060
D-glucose	Sigma-Aldrich	Cat#G7528
Kynurenic acid	Sigma-Aldrich	Cat#K3375
HEPES	Sigma-Aldrich	Cat#H3375
NaOH	Sigma-Aldrich	Cat#06203
K-gluconate	Sigma	Cat#G-4500
D-gluconic acid	Sigma-Aldrich	Cat#G1951

(*Continued on next page*)

Continued

REAGENT or RESOURCE	SOURCE	IDENTIFIER
CsOH	Sigma-Aldrich	Cat#516988
MgATP	Sigma-Aldrich	Cat#A9187
Na_2GTP	Sigma-Aldrich	Cat#G8877
K-Lucifer Yellow	Sigma-Aldrich	Cat#L0144
KOH	Sigma-Aldrich	Cat#P5958
$BaCl_2$	Sigma-Aldrich	Cat#B0750
BAPTA	Sigma-Aldrich	Cat#A4926
Tetrodotoxin	Hello Bio	Cat#HB1035
Nortriptyline	Sigma-Aldrich	Cat#N7261
VU 013992	Tocris	Cat#6877/10
ML 133 hydrochloride	Tocris	Cat#4549
Gabazine	Hello Bio	Cat#HB0901
Potassium ionophore I (Cocktail A)	Sigma-Aldrich	Cat#99311
N,N-Dimethyltrimethylsilylamine	Sigma-Aldrich	Cat# 226289
Paraformaldehyde	Fisher Scientific	Cat#P/0840/53
Goat serum	Sigma-Aldrich	Cat#G9023
Triton X 100	Sigma-Aldrich	Cat#T9284
EdU (5-ethynyl-2'-deoxyuridine)	Life Technologies	Cat#E10187
Alexa Fluor 555 Azide, Triethylammonium Salt	ThermoFisher Scientific	Cat#A20012
Alexa Fluor 647 Azide, Triethylammonium Salt	ThermoFisher Scientific	Cat#A10277
DAPI (4',6-Diamidino-2-phenylidole dihydrochloride)	Sigma-Aldrich	Cat#D9542
Fluoromount-G	SouthernBiotech	Cat#0100-01
TrypLE	ThermoFisher Scientific	Cat#12604013
Papain from papaya latex,buffered aqueous suspension, 2x Crystallized, 16-40 units/mg protein	Sigma-Aldrich	Cat#P3125
L-cysteine	Sigma-Aldrich	Cat#C7352
Trypsin inhibitor from Glycine max (soybean)	Sigma-Aldrich	Cat#T9003
Bovine Serum Albumin	Sigma-Aldrich	Cat#A4919
Deoxyribonuclease I from bovine pancreas,Type IV	Sigma-Aldrich	Cat#D5025
Poly-D-Lysine	ThermoFisher Scientific	Cat#A3890401
Matrigel	BD Bioscience	Cat#354230
Neurobasal medium	Gibco	Cat#21103049
DMEM	Invitrogen	Cat#41966029
B27 supplement	Gibco	Cat#17504001
Penicillin/Streptomycin	Sigma	Cat#P0781
Fetal bovine serum	Sigma	Cat#F7524
AraC	Sigma	Cat#C6645
N2 supplement	Made in house	N/A
Recombinant human Insulin-zinc	Gibco	Cat#12585-014
apo-Transferrin	Sigma-Aldrich	Cat#T1147
SOS	Cell Guidance Systems	Cat#M09-50
BDNF	Qkine	Cat#QK050
bFGF	PeproTech	Cat#100-18B
PDGF-AA	PeproTech	Cat#100-13A
Clozapine N-oxide(CNO)	Hello Bio	Cat#HB1807
Tamoxifen	Sigma	Cat#T5648

(*Continued on next page*)

Continued

REAGENT or RESOURCE	SOURCE	IDENTIFIER
Critical commercial assays		
A2B5 MicroBead Kit, human, mouse, rat	Miltenyi Biotec	Cat#130-093-392
Click-iT Cell Reaction Buffer Kit	ThermoFisher Scientific	Cat#C10269
Lipofectamine 3000 Transfection Reagent	ThermoFisher Scientific	Cat#L3000001
Experimental models: Organisms/strains		
Mouse: NG2-EYFP	Jacqueline Trotter; Karram et al.[42]	N/A
Mouse: Pdgfrα-CreER^{T2}	William D. Richardson; Rivers et al.[75]	N/A
Mouse: *R26*$^{Kir2.1\text{-}mCherry}$	Guillermina López-Bendito; Moreno-Juan et al.[60]	N/A
Mouse: Rosa26-tdTomato	Madisen et al.[61]	JAX Cat#007909; RRID:IMSR_JAX:007909
Mouse: Fucci2a	Mort et al.[76]	IMSR Cat# RBRC06511; RRID:IMSR_RBRC06511
Rat: Sox10-DsRed	Chen et al.[77]	N/A
Rat: Crl:CD(SD)	Charles River Laboratories	RRID:RGD_734476
Recombinant DNA		
Plasmid: Kcnj10 (NM_001039484) Mouse Tagged ORF Clone	OriGene	Cat#MG227234
Plasmid: pMAX-GFP	Lonza	N/A
Software and algorithms		
pClamp	Molecular Devices	https://www.moleculardevices.com/products/axon-patch-clamp-system/acquisition-and-analysis-software/pclamp-software-suite; RRID:SCR_011323
LAS AF/LAS X	Leica	https://www.leica-microsystems.com/products/microscope-software/details/product/leica-las-x-ls/; RRID:SCR_016555
FIJI	NIH	https://fiji.sc/ or; RRID:SCR_002285
Matlab	The MathWorks	https://uk.mathworks.com/; RRID:SCR_001622
Membrane capacitance and resistance script	Spitzer et al.[21]	N/A
GraphPad Prism	GraphPad Software	https://www.graphpad.com/scientific-software/prism/; RRID:SCR_002798

EXPERIMENTAL MODEL AND STUDY PARTICIPANT DETAILS

Experiments were performed in accordance with the EU guidelines for the care and use of laboratory animals, and with the guidelines of the UK Animals (Scientific Procedures) Act 1986 and subsequent amendments. Use of animals in this project was approved by the Animal Welfare and Ethical Review Body for the University of Cambridge and carried out under the terms of UK Home Office Licenses PP4353554, P9B1FBC4B and 70/7715. All mice were maintained under a 12 h light:12 h dark cycle with food and water supplied *ad libitum*. For electrophysiological studies, mice were heterozygous knock-in mice expressing EYFP under the endogenous NG2 promoter (NG2-EYFP mice) bred on a C57BL/6 background allowing for identification of OPCs by EYFP expression.[42] The ages of mice used were from P6 to P210 as stated in the text and figures. To increase the inward K^+ conductance in OPCs we crossed Cre-dependent *R26*$^{Kir2.1\text{-}mCherry}$ mice,[60] with PDGFRα-CreER^{T2} mice[75] to generate PDGFRα-CreER^{T2}:R26$^{Kir2.1\text{-}mCherry}$ mice, referred to as Kir2.1 mice. Following tamoxifen administration, OPCs in these mice overexpress Kir2.1 channels and are labelled with mCherry. Tamoxifen was given by intraperitoneal injection where concentration was matched to the age of the animal: P1-3 (25 mg/kg), P13 (35 mg/kg) and over P14 (50 mg/kg). Wildtype littermates were used as controls, except for electrophysiological experiments where we crossed PDGFRα-CreER^{T2} mice with the Cre-dependent reporter line Rosa26-tdTomato[61] (Jax mice #007909) and administered 35 mg/kg-50 mg/kg to pups by intraperitoneal injection, as above, or 150 mg/kg tamoxifen by oral gavage at P21-23. To assess electrophysiological properties of different cell cycle states in OPCs we crossed fluorescent ubiquitination-based cell-cycle indicator 2a (Fucci2a, kindly provided by Richard Mort) mice, which express fluorescent indicators for different cell cycle phases (Figure 5A),[76] to the NG2-EYFP mice to generate NG2-EYFP:Fucci2a mouse line. For all mouse lines, both male and female mice were used.

METHOD DETAILS

Brain slices preparation

Brain slices were prepared as described previously.[21,78,79] Briefly, acute brain slices (225 μm thick) were prepared from the forebrain (coronally cut) from transgenic mice in ice-cold (~3°C) oxygenated (95% O_2–5% CO_2) artificial cerebrospinal fluid (aCSF) solution and kept at room temperature (RT) for a 1h recovery period. aCSF contained (in mM): 126 NaCl, 24 $NaHCO_3$, 1 NaH_2PO_4, 2.5 KCl, 2.5 $CaCl_2$, 2 $MgCl_2$, 10 D-glucose, pH 7.4. Kynurenic acid (1 mM) was added to block glutamate receptors, which might be activated during the dissection procedure.

Solutions

Solutions were prepared as described previously.[21,78,79] Briefly, acute brain slices were superfused at RT with HEPES-buffered aCSF, at a rate of 2 ml/min, containing (in mM): 144 NaCl, 2.5 KCl, 10 HEPES, 1 NaH_2PO_4, 2.5 $CaCl_2$, 10 glucose, pH set to 7.35 with NaOH, continuously bubbled with 100% O_2; 2 mM $MgCl_2$ was added in some experiments as stated in the text and figures. OPCs and cortical neurons were whole-cell patch clamped with electrodes containing an intracellular solution comprised (in mM) of either 130 K-gluconate or 130 Cs-gluconate, 4 NaCl, 0.5 $CaCl_2$, 10 HEPES, 10 BAPTA, 4 MgATP, 0.5 Na_2GTP, 2 K-Lucifer yellow, pH set to 7.3 with KOH (or with CsOH). Final osmolality was ~290 mOsm/kg. HEPES-buffered aCSF solution without Mg^{2+} ions was used to elicit neuronal activity in the cortex by disinhibiting NMDA receptors.[47] Bath applied 200 μM $BaCl_2$ was used to block passive K^+ conductance, 1 μM TTX was used to block neuronal activity, and 300 μM nortriptyline was used to block Kir channels.[52] 30 μM VU0134992 was used to selectively block Kir4.1 channels[53] and 30 μM ML133 hydrochloride was used to selectively block Kir2.1 channels.[54] Gabazine 10 μM together with 0 Mg^{2+} aCSF was used to trigger spontaneous neuronal activity in the hippocampus.[51]

Electrophysiology

For whole-cell patch clamp experiments parenchymal $EYFP^+$ OPCs were selected in the brain area of interest ($EYFP^+$ cells located close to or on blood vessels were excluded). For OPCs recorded in Kir2.1 mice and tdTomato reporter mice, OPCs were selected based on their morphology and typical electrophysiological profile.[79] When unspecified, cortical OPCs were mainly recorded in cortical layers 2-5 of the somatosensory cortex, and some in the cingulate and motor cortex, and OPCs in the corpus callosum were in the anterior corpus callosum. Patched cells were confirmed as OPCs by their post-recording dye-fill morphology, which overlaid EYFP, and by post-hoc antibody labelling against the proteoglycan NG2 to identify oligodendrocyte precursors (Figures 1A–1C) or against GFP/EYFP, as described previously.[21,78] Recording electrodes had a resistance of 2.5–6 MΩ and the uncompensated series resistance was 32 ± 1 MΩ. Inclusion criteria was based on series resistance, leak current being lower than 500 pA and a stable baseline. Electrode junction potential (-14 mV) was compensated. A Multiclamp 700B or Axopatch 200 (both Molecular Devices) or EPC-7 (HEKA) were used for voltage-clamp data acquisition. Data were sampled at 50 kHz and filtered at 10 kHz using pClamp10.3, 10.7, 11.0 or 11.2 (Molecular Devices). Series and membrane resistance together with membrane capacitance were analysed using custom-written MATLAB scripts (MathWorks), as previously described.[21,79] Membrane potential and inward conductance determined from the best fit of the linear part of the slope of the inward currents analysed between -154 mV and the equilibrium potential, a measure of inward conductance, were calculated in Excel.

To calculate the current evoked by changing the $[K^+]_e$ in the aCSF (ΔI) we used a variation of the Nernst equation, where Rm is membrane resistance, V_h is holding potential, R is gas constant, T is absolute temperature, F is Faraday constant, $[K^+]_e$ and $[K^+]_i$ are external and internal K^+ concentrations. If the $[K^+]_e$ changes, the difference between the resting and the evoked currents will be:

$$\Delta I = \frac{1}{R_m}\left(V_h - \frac{RT}{F}\ln\frac{[K^+]_e}{[K^+]_i}\right) - \frac{1}{R_m}\left(V_h - \frac{RT}{F}\ln\frac{[K^+]'_e}{[K^+]_i}\right) = \frac{1}{R_m}\frac{RT}{F}\ln\frac{[K^+]'_e}{[K^+]_e}$$

where $[K^+]'_e$ is the new $[K^+]_e$.

Fabrication of K^+-selective microelectrodes and measurement of extracellular K^+ concentration

Single-barrelled K^+-selective microelectrodes (K^+-ISMs) were prepared using thin-walled borosilicate capillaries (GC150T-7.5, Harvard Apparatus, MA). The interior walls of the capillaries were silanized by silan vapours using *N,N*-Dimethyltrimethylsilylamine at 200°C for 60 minutes. The tips (about 2 μm in diameter) of K^+-ISMs were filled with a small quantity of the liquid ion-exchanger Potassium ionophore I (Cocktail A) using capillary forces, and the above part of the electrode was backfilled with 200 mM KCl. The reference electrode, which recorded local field potentials, was made of standard patch clamp glass capillaries (GC150F-10, Harvard Apparatus, MA) and filled with 1M NaCl. Both electrodes were connected by a chlorinated silver wire to the input of the amplifier. The K^+-ISMs were calibrated using solutions containing 1, 2, 10, and 20 mM KCl. K^+-ISMs were tested for a Nernstian response (>50 mV) to a 10-fold increase in $[K^+]$, at the beginning and the end of the experiment. Data were acquired using Axopatch 700B amplifier, sampled at 20 kHz, low pass filtered (2 kHz), digitized (Digidata 1440), and stored and analyzed using pCLAMP 10.7 software (all Molecular Devices). The signal from the reference electrode was offline subtracted from the signal at the K^+-ISM to obtain a signal proportional to actual K^+ concentration. The relationship between the measured voltage and the actual K^+ concentration was derived from the log-linear fit function in Excel.

Immunohistochemistry

Following transcardial perfusion with 4% paraformaldehyde (PFA), 50-100 μm thick brain slices were cut using a vibratome. For immunohistochemistry following patch clamp, brain slices were postfixed in PFA for 1 hour at RT. Antibody labelling was performed as described previously.[21,80] Briefly, slices were incubated in permeabilization and blocking solution (10% goat serum (GS), 0.5% Triton X-100, in PBS) for 4-6 hours. Slices were incubated with primary antibodies in PBS overnight at RT. After washing, slices were incubated with secondary antibodies in PBS for 4 hours at RT or overnight at 4°C. Following washing and DAPI staining for cell nuclei, slices were mounted (Fluoromount) and imaged on a confocal microscope (Leica SP5, SP8 or Stellaris). To quantify cell proliferation, 0.2 mg/ml 5-ethynyl-2'-deoxyuridine (EdU) was administered in the drinking water or 25 mg/kg EdU was administered by intraperitoneal injection. EdU was detected using a Click It kit (Thermofisher) according to the manufacturer's instructions, and visualized with Alexa Fluor-conjugated azides. For cell number quantification, at least 2 z-stacks per area were taken in each brain slice, in at least 2 slices per animal. Quantification was performed manually blinded using FIJI.

For the detection of EYFP we used chicken anti-GFP antibody (1:500 or 1:1000); NG2 Chondroitin Sulfate Proteoglycan was detected using rabbit anti-NG2 antibody (Cat#AB5320, Millipore, 1:300); neuronal cell bodies were visualized by rabbit anti-NeuN (1:200) or mouse anti-NeuN antibody (1:300); mCherry was detected by rabbit anti-mCherry (1:700) or chicken anti-mCherry antibody (1:500); proliferating cells were labelled with rabbit anti-Ki67 (1:300).

Increasing neuronal activity by DREADDs

Adult mice (24-26-week-old NG2-EYFP mice, 3-7 mice per group for electrophysiology and 8 mice per group for immunohistochemistry; 8-9-week old Kir2.1 mice, 4 mice per group for electrophysiology, 6-7 mice per group for immunohistochemistry) were anesthetized with 2% Isoflurane and injected stereotaxically into the somatosensory cortex (antero-posterior at bregma, mediolateral 2.25 mm, dorso-ventral 1.4 mm, 200 nl/min) with 500 nl of pAAV8-hSyn-hM3D(Gq)-mCherry (Gq) and pAAV8-hSyn-mCherry (control) viruses, or pAAV5-SYN1-HA-hM3D(Gq)[74] and pAAV5-hSyn-EGFP (control), (all from Addgene). Meloxicam was given pre-operatively and for 3 days post injection for pain relief. The mice were left at least 3 weeks after the injection to ensure enough gene expression. To activate Gq and increase neuronal activity, CNO (HelloBio) was administered at 25 mg/L in the drinking water of both Gq and control mice.

Neuron-OPC co-cultures

To prepare cortical neuron cultures, cortices were dissected from E19-20 CD rat embryos and cells were dissociated with TrypLE. 100K cells were plated on poly-D-lysine coated coverslips in 24-well plates, in neurobasal medium supplemented with B27, 5% fetal bovine serum, and 1% penicillin/streptomycin. A half-medium change (without fetal bovine serum) was performed on day 4, along with 1 μM AraC treatment. Following this, half-media changes were performed twice per week, until OPCs were added at 14 days *in vitro* (DIV) and the medium was changed to neurobasal supplemented with 0.5X N2 (made in house), 0.5X B27 and 20 ng/ml BDNF.

OPCs were isolated from P0-2 Sox10-DsRed rats[77] after tissue digestion by Worthington Papain solution with DNaseI, using magnetic cell sorting, with microbeads conjugated to anti-A2B5, following the manufacturer's instructions and as described previously.[79] 5-20K OPCs were plated in 50 μl volume on poly-D-lysine coated coverslips in 24-well plates in neurobasal medium supplemented with 0.5X N2 and 0.5X B27, or plated onto 100K cortical neuron cultures. 20 ng/ml BDNF was added every 2nd day and 10 μg/ml Insulin-zinc was added daily. OPCs were used for electrophysiological recordings on 4-5 DIV.

To assess proliferation, 400 nM EdU was added to the medium for 24 hours on 4 DIV. For antibody staining, coverslips were fixed in 4% PFA for 10 minutes, and following washes, they were blocked and permeabilized for one hour at RT in 10% GS, 0.1% Triton X 100, then incubated with rabbit anti-NG2 (Cat#ab275024, Abcam, 1:100) antibody overnight at 4°C. Coverslips were washed thrice in PBS and incubated with secondary antibody (1:1000) for two hours at RT. Following two PBS washes, coverslips were incubated with DAPI. EdU was detected using a Click It kit according to the manufacturer's instructions. Following washing, coverslips were mounted (Fluoromount) on glass slides and imaged with a Leica TCS SP8 confocal.

Kir4.1 channel overexpression in OPC cultures

Mixed glial cultures were prepared from P0-2 CD rats as previously described.[81] Briefly, OPCs were isolated from mixed glial cultures by modified shake off method of McCarthy and de Vellis.[82,83] OPCs were resuspended in OPC medium containing DMEM with 1% penicillin/streptomycin, 5 μg/ml human recombinant Insulin/zinc, 50 μg/ml apo-Transferrin, 2% SOS,[84] and supplemented daily with 10 ng/ml human bFGF and 10 ng/ml PDGF-AA. OPCs were plated in OPC medium on PDL-coated 6-well plates (100,000 cells/well) or on PDL and matrigel coated glass cover slips (22 mm diameter, 50K cells/cover slip). OPCs were transfected with 5 μg Kir4.1-GFP (OriGene) or GFP control plasmid (Lonza) using Lipofectamine 3000 transfection reagent and used for experiments 24-48hrs after lipofection.

To assess proliferation, coverslips were fixed in 4% PFA, and following PBS washes, they were blocked and permeabilized for one hour at RT in 10% GS, 0.5% Triton X100, then incubated with rabbit anti-Ki67 (Abcam, 1:300) overnight at 4°C. Coverslips were washed thrice in PBS and incubated with secondary antibody (1:500) for one hour at RT. Following three PBS washes, coverslips were incubated with DAPI, washed and mounted (Fluoromount) on glass slides and imaged with a Leica SP5 confocal.

QUANTIFICATION AND STATISTICAL ANALYSIS

All statistical analyses were performed in GraphPad Prism, or manually calculated. Data are shown as mean ± s.e.m, and the number of experiments indicated in text or figures, as appropriate. Where relevant, normality of data was assessed using Shapiro–Wilk tests. Significance in the variance between data set was assessed using F-test or Brown–Forsythe test and to directly test difference in variance, Levene's variability test was used. One-way ANOVA, followed by Holm–Bonferroni-corrected post-hoc t-tests were used to compare multiple groups. Two-way ANOVA with Šídák's multiple comparisons tests were used to assess the significant differences between current/voltage (I/V) curves at different ages. Paired or unpaired two-tailed Student's t-test was used for comparison of means between two groups. In cases where variances were unequal, Welch's correction was used for two-tailed t-tests and one-way ANOVA. Gaussian curve fits were evaluated by extra-sum of squares F-test and Akaike's information criteria. Differences in percentages between age groups were analysed by Chi-squared test with Yates correction and distribution test. Correlation between two parameters was assessed by linear regression analyses.

Cell Reports, Volume 43

Supplemental information

Heterogeneity in oligodendrocyte precursor cell proliferation is dynamic and driven by passive bioelectrical properties

Helena Pivoňková, Sergey Sitnikov, Yasmine Kamen, An Vanhaesebrouck, Moritz Matthey, Sonia Olivia Spitzer, Yan Ting Ng (吳胤霆), Chenyue Tao (陶辰玥), Omar de Faria Jr., Balazs Viktor Varga, and Ragnhildur Thóra Káradóttir

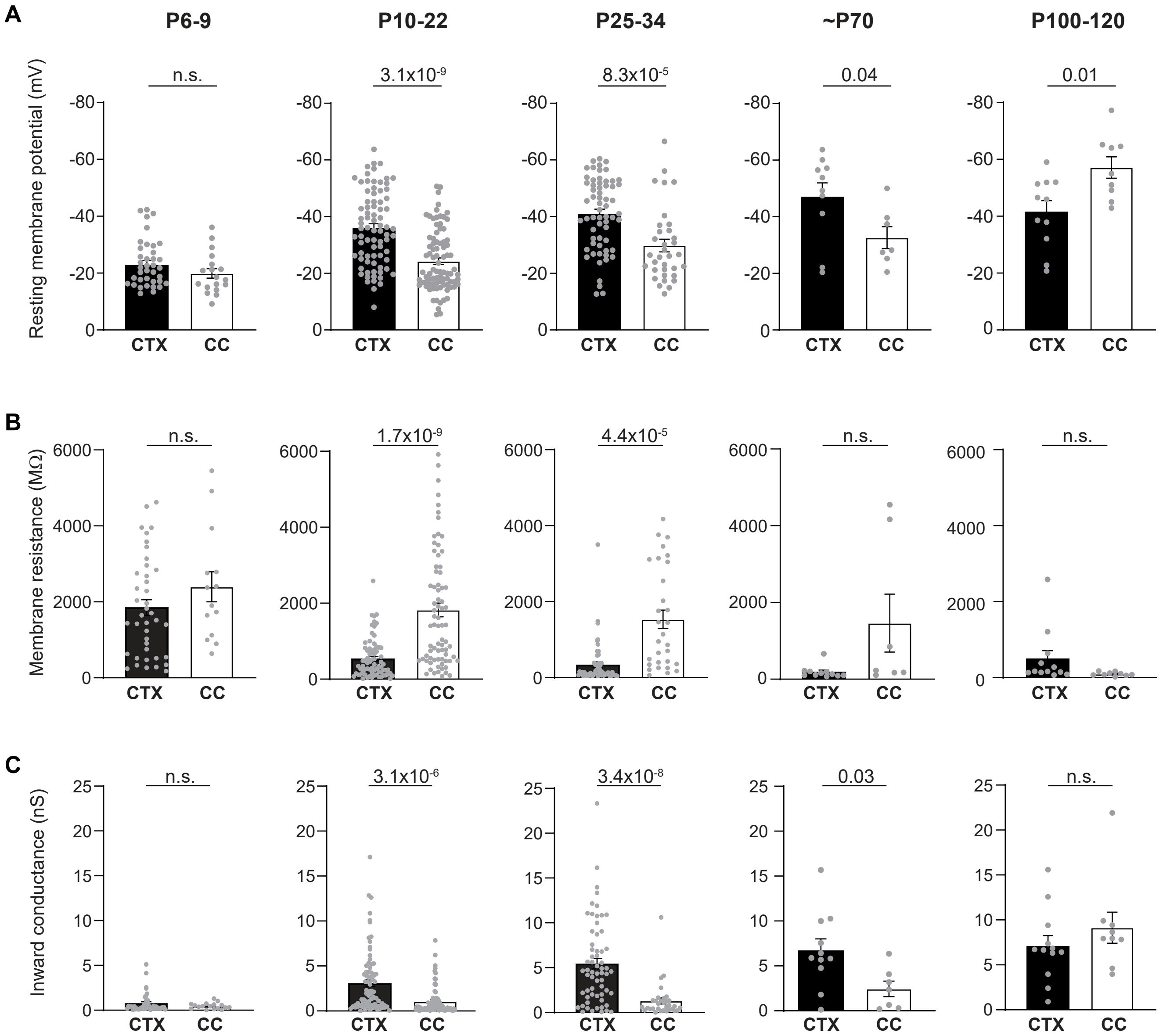

Figure S1. Membrane properties comparison between cortical and callosal OPCs at different ages, related to Figure 1.

(**A**) Resting membrane potential, (**B**) membrane resistance and (**C**) inward conductance are compared between cortical and callosal OPCs at different ages.

Individual grey dots indicate individual recorded cells. p values were calculated using unpaired two-tailed t-tests or t-tests with Welch's correction. Values are given as mean ± s.e.m..

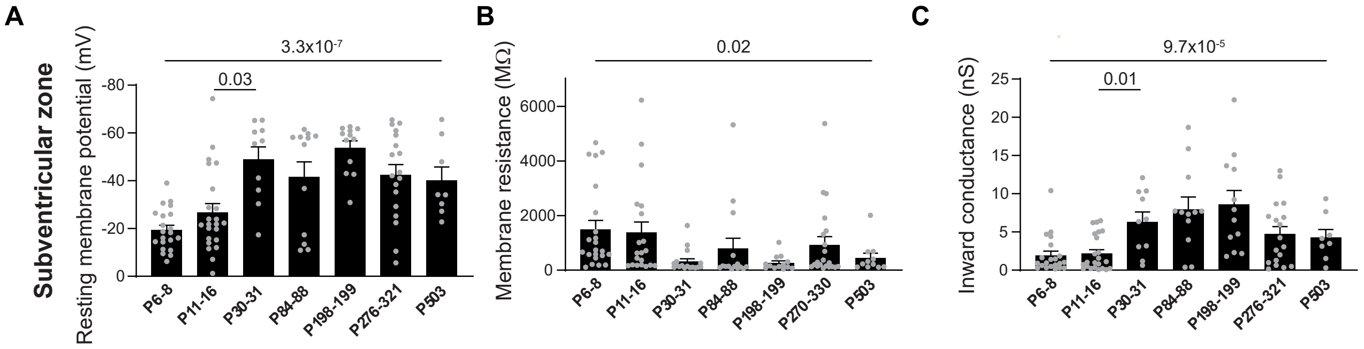

Figure S2. Membrane properties of OPCs in the subventricular zone, related to Figure 1.

(**A**) Resting membrane potential, (**B**) membrane resistance and (**C**) inward conductance were recorded in $EYFP^+$ cells in NG2-EYFP mice in the subventricular zone at different ages.

Grey dots represent individual recorded cells. p values were calculated using one-way ANOVA (top) with Holm-Bonferroni post-hoc tests (bottom). Values are given as mean ± s.e.m..

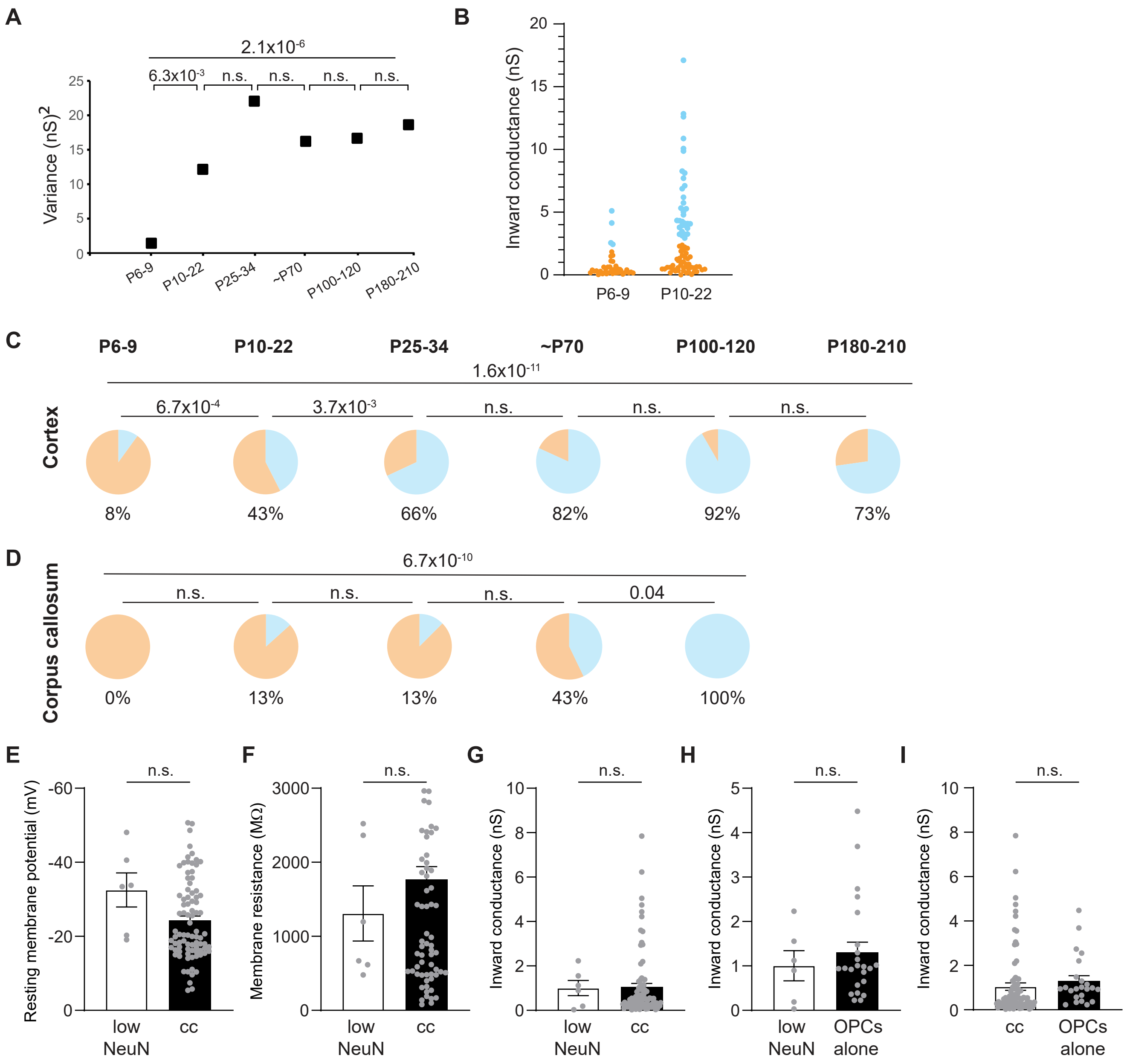

Figure S3. OPC subgroups based on inward conductance variability, related to Figure 2.

(**A**) Variance in cortical OPC inward conductance at different ages. p values of the differences in the variability between age groups were calculated using Levene's variability multi-comparison test.

(**B**) Inward conductance in OPCs in young animals clearly show separation into two groups, OPCs with low conductance (orange) and OPCs with high conductance (blue), the number of which increases with age.

(**C-D**) Percentages of OPCs with low (orange) and high (blue) inward conductance in different age groups in the cortex (**C**) and corpus callosum (**D**). OPCs were assigned into high conductance group when the inward conductance was higher than 2.35 nS as calculated from the 90th percentile of the two Gaussian curves (see the results and Figure 2). The percentages below the pie charts indicate the proportion of OPCs with high inward conductance. p values were calculated with χ^2- test (top) and χ^2- test with Yates correction (bottom).

(**E-I**) OPCs in areas of low neuronal density have similar membrane properties. The resting membrane potential (**E**), membrane resistance (**F**), and inward conductance (**G**) in cortical OPCs in areas of low neuronal density are similar to age-matched passive membrane properties of OPCs in the corpus callosum. OPCs in low neuronal density cortical areas and in the corpus callosum have similar inward conductance to OPCs cultured without neurons (**H,I**).

Dots represent individual recorded cells. p values are from two-tailed unpaired t-test or t-test with Welch's correction. Data are presented as mean ± s.e.m..

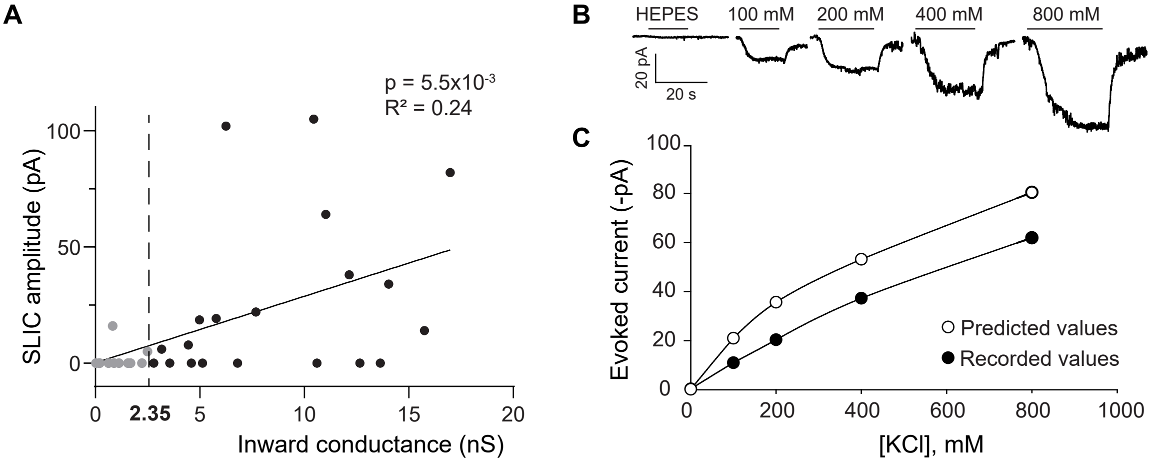

Figure S4. OPCs respond to increases in $[K^+]_e$ by increasing the inward current, related to Figure 4.

(**A**) The amplitude of SLICs correlate with the inward conductance in OPCs. Dots represent individual recorded cells. p value is from linear regression analysis.

(**B**) Increasing the concentration of K^+ in aCSF leads to proportional increase in the inward current in OPCs.

(**C**) The recorded inward current in OPCs evoked by increasing concentration of K^+ in aCSF follows the current predicted from the Nernst equation (see Methods).

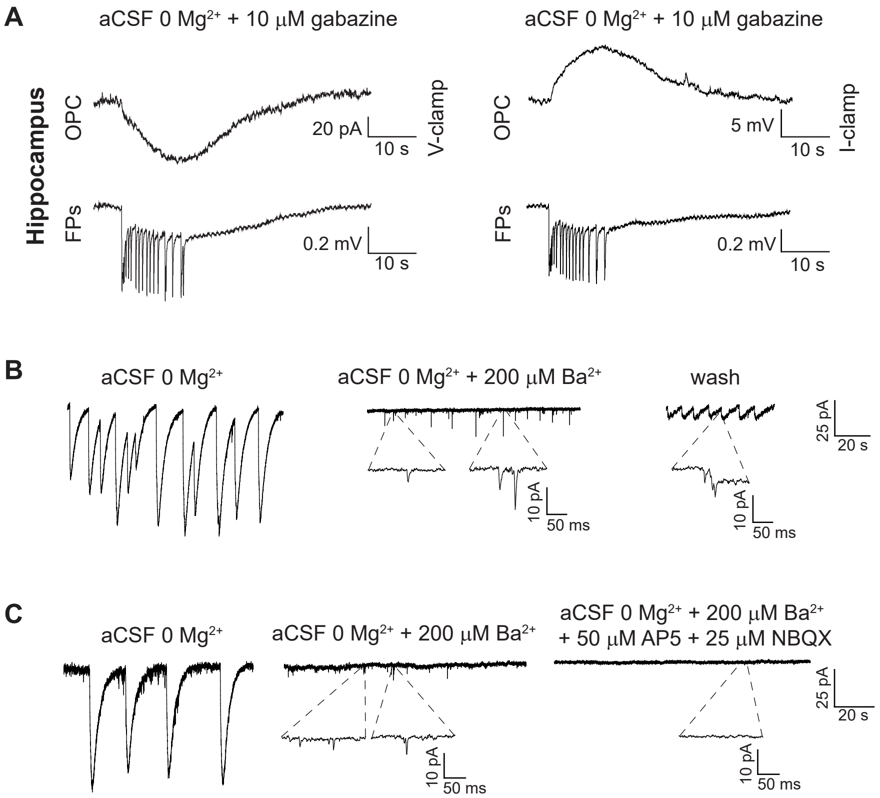

Figure S5. SLICs properties in hippocampus and in the cortex, related to Figure 4.

(**A**) Representative traces of an OPC recorded in the stratum radiatum of the hippocampus in voltage-clamp and current-clamp mode together with simultaneous recording of neuronal activity using extracellular field potentials (FPs). High neuronal activity was evoked by using 0 Mg^{2+}/gabazine (10 µM) aCSF. SLICs are accompanied by simultaneous depolarisations in OPCs.

(**B**) Ba^{2+} ions blocked SLICs completely, however, they did not affect spontaneous excitatory postsynaptic currents (EPSCs) in OPCs showing that neuronal activity and synaptic activity is preserved in these conditions. The effect of Ba^{2+} ions on SLICs was partially reversible.

(**C**) EPSCs in OPCs were completely blocked by 50 µM AP5 and 25 µM NBQX.

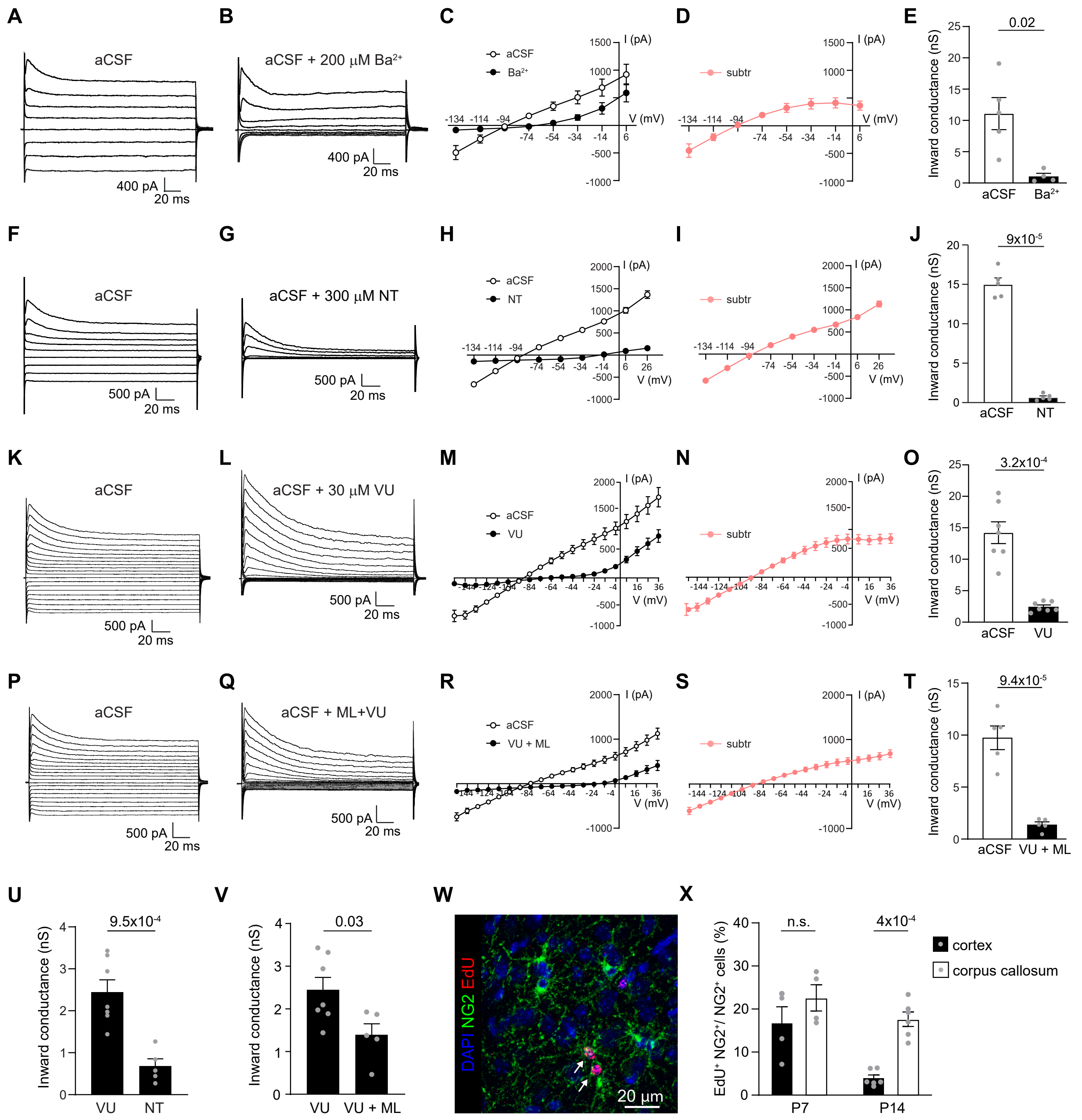

A
aCSF
400 pA
20 ms
B
aCSF + 200 µM Ba2+
400 pA
20 ms
C
aCSF
Ba2+
I (pA)
V (mV)
D
subtr
I (pA)
V (mV)
E
0.02
Inward conductance (nS)
aCSF
Ba2+
F
aCSF
500 pA
20 ms
G
aCSF + 300 µM NT
500 pA
20 ms
H
aCSF
NT
I
subtr
J
9x10-5
aCSF
NT
K
aCSF
500 pA
20 ms
L
aCSF + 30 µM VU
500 pA
20 ms
M
aCSF
VU
N
subtr
O
3.2x10-4
aCSF
VU
P
aCSF
500 pA
20 ms
Q
aCSF + ML+VU
500 pA
20 ms
R
aCSF
VU + ML
S
subtr
T
9.4x10-5
aCSF
VU + ML
U
9.5x10-4
VU
NT
V
0.03
VU
VU + ML
W
DAPI NG2 EdU
20 µm
X
EdU+ NG2+/ NG2+ cells (%)
n.s.
4x10-4
cortex
corpus callosum
P7
P14

Figure S6. Inward currents are mediated by K^+ conductance through Kir4.1 and Kir2.1 channels and correlate with OPC proliferation potential, related to Figure 4 and 6.

(**A,F,K,P**) Membrane currents evoked by stepping the membrane potential of recorded cells from -134 mV to +26 mV in 20 mV voltage steps (A,F), or from -154 mV to +36 mV in 10 mV voltage steps (K,P), in mouse cortical OPCs in control conditions.

(**B,G,L,Q**) Membrane currents evoked in OPCs after bath application of 200 µM Ba^{2+} (non-specific K^+ channel blocker), 300 µM nortriptyline (NT; inward rectifying K^+ channel blocker), 30 µM VU0134992 (a specific Kir4.1 channel blocker), and a combination of 30 µM VU0134992 + 30 µM ML133 hydrochloride (specific Kir4.1 and Kir2.1 channel blockers, respectively).

(**C,H,M,R**) Current/voltage (I/V) graphs of OPC membrane currents in control conditions and after application of different K^+ channel blockers.

(**D,I,N,S**) Current/voltage (I/V) graphs of the subtracted currents between the control conditions and after application of different K^+ channel blockers.

(**E,J,O,T**) Inward conductance in OPCs is blocked by different K^+ channel blockers.

(**U and V**) 30 µM VU alone does not block the inward K^+ currents completely, but 300 µM nortriptyline or the combination of 30 µM VU0134992 + 30 µM ML133 hydrochloride do block the currents entirely.

(W) Representative image of NG2 and EdU labelling in the cortex of a P14 mouse. White arrows indicate $NG2^+EdU^+$ cells.

(X) Mice were injected with 25 mg/kg EdU at P6 or P13 and perfused-fixed 24 hours later, at P7 or P14. At P7, before the onset of high inward conductance in the cortex, OPCs in the cortex and corpus callosum proliferate at similar rates. At P14, after the onset of high inward conductance in cortical OPCs, proliferation is reduced in cortical OPCs compared to callosal OPCs.

Dots represent individual recorded cells (E, J, O, T, U, V) or animals (X). p values are from two-tailed unpaired t-test or t-test with Welch's correction (E, J, O, T, U, V) or from two-way ANOVA with Šídák's multiple comparisons test (X). Data are presented as mean ± s.e.m..

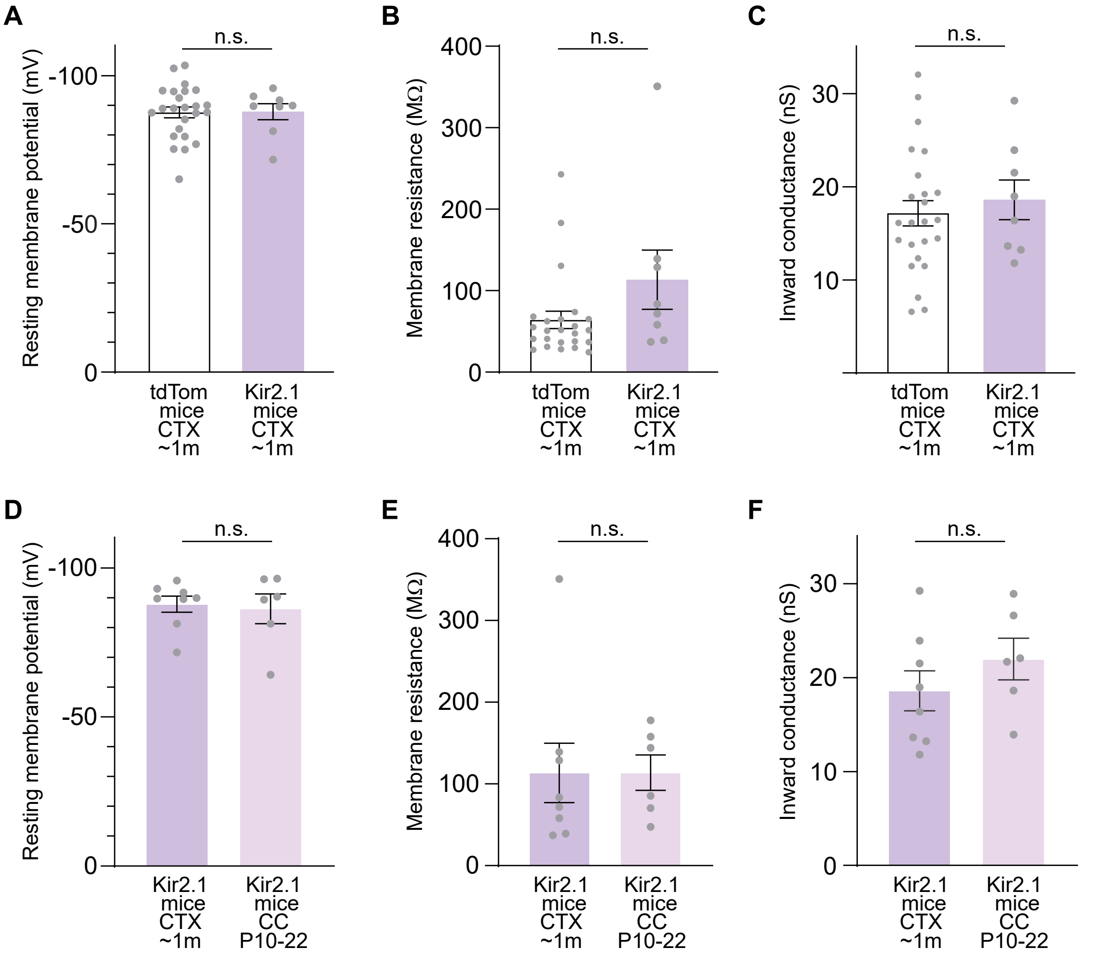

Figure S7. OPCs in Kir2.1 mice have physiological membrane properties and lose regional differences, related to Figure 6.

(**A**) The resting membrane potential, (**B**) membrane resistance, (**C**) and inward conductance in cortical (P25-30) OPCs overexpressing Kir2.1 channels are comparable to these passive membrane properties in age-matched wild type mice.

(**D**) The resting membrane potential, (**E**) membrane resistance, (**F**) and inward conductance in cortical and callosal OPCs overexpressing Kir2.1 channels in young animals are comparable.

Dots represent individual recorded cells. p values are from two-tailed unpaired t-test or t-test with Welch's correction. Data are presented as mean ± s.e.m..
